# Multifunctional polymeric micelles for delivery of drugs and siRNA

**DOI:** 10.3389/fphar.2014.00077

**Published:** 2014-04-25

**Authors:** Aditi M. Jhaveri, Vladimir P. Torchilin

**Affiliations:** Department of Pharmaceutical Sciences, Center for Pharmaceutical Biotechnology and Nanomedicine, Northeastern UniversityBoston, MA, USA

**Keywords:** micelles, multifunctional, nanocarriers, polymeric, siRNA, targeted

## Abstract

Polymeric micelles, self-assembling nano-constructs of amphiphilic copolymers with a core-shell structure have been used as versatile carriers for delivery of drugs as well as nucleic acids. They have gained immense popularity owing to a host of favorable properties including their capacity to effectively solubilize a variety of poorly soluble pharmaceutical agents, biocompatibility, longevity, high stability *in vitro* and *in vivo* and the ability to accumulate in pathological areas with compromised vasculature. Moreover, additional functions can be imparted to these micelles by engineering their surface with various ligands and cell-penetrating moieties to allow for specific targeting and intracellular accumulation, respectively, to load them with contrast agents to confer imaging capabilities, and incorporating stimuli-sensitive groups that allow drug release in response to small changes in the environment. Recently, there has been an increasing trend toward designing polymeric micelles which integrate a number of the above functions into a single carrier to give rise to “smart,” multifunctional polymeric micelles. Such multifunctional micelles can be envisaged as key to improving the efficacy of current treatments which have seen a steady increase not only in hydrophobic small molecules, but also in biologics including therapeutic genes, antibodies and small interfering RNA (siRNA). The purpose of this review is to highlight recent advances in the development of multifunctional polymeric micelles specifically for delivery of drugs and siRNA. In spite of the tremendous potential of siRNA, its translation into clinics has been a significant challenge because of physiological barriers to its effective delivery and the lack of safe, effective and clinically suitable vehicles. To that end, we also discuss the potential and suitability of multifunctional polymeric micelles, including lipid-based micelles, as promising vehicles for both siRNA and drugs.

## Introduction

Nanotechnology is currently at the forefront of drug delivery research, and has provided innovative platforms for management of diseases like cancer, which pose a significant challenge for researchers and patients alike. A variety of nanoscale systems including polymeric and metallic nanoparticles, liposomes, polymeric micelles, nanogels, nanocapsules, dendrimers, carbon nanotubes, nanocrystals and solid lipid nanoparticles, are currently under active investigation for delivery of small molecule drugs as well as therapeutic macromolecules like proteins, peptides, aptamers, DNA and small interfering RNA (siRNA) (Torchilin, 2006; Peer et al., 2007; Wang et al., 2012a). These nanomedicines can successfully overcome many drawbacks of free drugs and therapeutic molecules which include but are not limited to poor solubility, non-selective activity, poor biodistribution and pharmacokinetics (PK), dose-limiting toxicity and also multi-drug resistance (Allen and Cullis, 2004; Jabr-Milane et al., 2008; Sawant et al., 2012). Some of the salient advantages of nanocarriers include their increased drug stability, ability to solubilize hydrophilic and hydrophobic agents, improved PK and biodistribution, tunable payload release, the ability to specifically target their payload to diseased tissues and cells by modification of their surface chemistries, and finally their ability to respond to various internal and external stimuli for “triggered” release to achieve temporal and spatial control over the release of therapeutic payloads (Torchilin, 2006; Peer et al., 2007; Duncan and Gaspar, 2011; Schroeder et al., 2012).

One has to keep in mind, however, that due to the inherent advantages of nanomedicines over conventional therapeutics, the rapid pace of development of nanocarriers and a paucity of detailed systemic toxicology studies on them, it is easy to overlook certain toxicity concerns. It is critical to appreciate that material properties differ significantly at the nanoscale range from those seen in the bulk, with greatly increased surface-to-volume ratios, altered surface chemistry and an increased chemical reactivity (Elsaesser and Howard, 2012). When designing nanocarriers, one needs to address the variables which may lead to potential safety concerns including the material used for construction of the nanocarriers, dose or concentration of the nanocarriers, their size, shape, surface charge, reactivity and solubility (Ai et al., 2011; Elsaesser and Howard, 2012). Due consideration of these variables could enable the development of robust nanosystems with many promising features.

Although most of the types of nanocarriers listed in this section are capable of providing many of the advantages already mentioned, for the purpose of this review, the discussion is specifically focused on one class of versatile nanocarriers, the polymeric micelles, which are core-shell nano-constructs formed by self-assembly of amphiphilic copolymers. Various modifications for polymeric and lipid-based micelles including those for passive targeting, active targeting and stimuli sensitive release, are discussed with recent examples from the literature. The focus then shifts to micelles which can combine multiple favorable features to form multifunctional polymeric micelles. Since the discovery of RNA interference (RNAi), and its ability to silence virtually any gene, substantial research efforts have been dedicated to the development of suitable carriers to deliver siRNA for cancer therapy (Fire et al., 1998; Shen et al., 2012). To that end, this review also discusses recent progress in siRNA delivery for cancer, challenges facing it and the role of multifunctional polymeric micelles. Finally, we discuss multifunctional micelles which can simultaneously deliver both small molecule drugs and siRNA to tumors.

## Polymeric micelles: background and relevance as therapeutic nanocarriers for cancer

**Figure 1:**
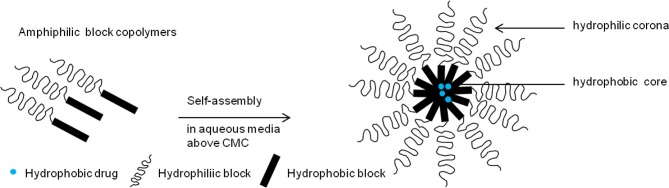
**Micelle formation**. Drug-loaded polymeric micelle formed from self -assembly of amphiphilic block copolymers in aqueous media.

Polymeric micelles are spherical, colloidal, supramolecular nano-constructs (10–100 nm) usually formed from the self-assembly of amphiphilic block copolymers which consist of both hydrophilic and hydrophobic units in an aqueous environment (Yokoyama et al., 1998; Jones and Leroux, 1999; Torchilin, 2001, 2007; Croy and Kwon, 2006). This self-assembly of amphiphilic monomers is entropically favored, and occurs above their critical micelle concentration (CMC) to result in the formation of micelles with a core-shell structure (Torchilin, 2001; Sutton et al., 2007). The hydrophobic portion of the block copolymer forms the core of micelles, while the hydrophilic portion forms the shell or the corona (Figure 1). Generally, micelles of amphiphilic copolymers with low CMC values exhibit greater stability even at low concentrations of the amphiphile in the medium. Increasing the hydrophobicity of the copoloymer reduces the CMC which in turn, increases the micelle stability (Torchilin, 2001; Kabanov et al., 2002). Non-polar molecules are solubilized within the hydrophobic core of micelles; polar molecules get adsorbed on the micelle surface, whereas molecules with intermediate polarity distribute along the surfactant molecules in intermediate positions.

Various amphiphilic copolymers including di-block (A-B), tri-block (A-B-A) as well as graft copolymers can be used to form micelles (Torchilin, 2001). By far, the most frequently utilized hydrophilic block for both di- and tri-block copolymers is poly(ethylene oxide) (PEO) also known as poly(ethylene glycol) (PEG). Other corona forming polymers like poly (N-vinyl pyrrolidone) (PVP) (Bailly et al., 2012) and poly (N-isopropyl acrylamide) (pNIPAAm) (Kim et al., 2013) have also been reported. A number of different core forming blocks have been reported to date which include poly(propylene oxide) (PPO) (Sharma et al., 2008), poly ε-caprolactone (PCL) (Kanazawa et al., 2013; Laouini et al., 2013), poly (L-lactide) (Xu et al., 2013a), poly(lactide-co-glycolic acid) (PLGA) (Koyamatsu et al., 2013), poly(L-aspartic acid) (pAsp) (Yokoyama et al., 1990; Kagaya et al., 2012), poly(L-histidine) (pHis) (Wu et al., 2013), poly (β-amino ester) (PbAE) (Min et al., 2010) and short, hydrophobic phospholipid residues like disteroyl phosphatidyl ethanolamine (DSPE) (Perche et al., 2012; Sawant et al., 2013a; Salzano et al., 2014).

Polymeric micelles exhibit several features that favor their utility for drug delivery applications in cancer. The most important advantage of polymeric micelles is their ability to solubilize poorly water soluble or hydrophobic drugs within their core, thus enhancing their bioavailability. A large number of high throughput screen (HTS) derived hits, lead molecules, development candidates and eventually, marketed drugs share the common characteristic of possessing high hydrophobicities and consequently, low aqueous solubilities (Williams et al., 2013). Even with sophisticated combinatorial chemistry approaches to generate large compound libraries and advanced HTS technologies, it has been difficult to obtain compounds with acceptable water solubility without compromising on their potency. About 40% of currently marketed drugs and up to 75% compounds currently under development have been suggested to be poorly water soluble (Di et al., 2009, 2012; Williams et al., 2013). Anti-cancer drugs, which are most often polycyclic compounds, also suffer the same fate. Moreover, because of the intrinsic hydrophobicity of many drugs, parenteral administration becomes problematic and undissolved drug aggregates can lead to embolization of blood capillaries (≤5 μm) before they reach the tumor (Fernandez et al., 2001). Low solubility coupled with drug excretion and metabolic degradation also results in poor systemic drug concentrations (Torchilin et al., 2003). Polymeric micelles can not only solubilize hydrophobic drugs, but can also protect them from inactivation in the biological milieu and thus increase their bioavailability (Torchilin, 2001). Another important advantage of polymeric micelles is their small size which allows them to circulate in the blood for extended periods by evading the mononuclear phagocytic system (MPS) in the liver. At the same time, their size is large enough to preclude fast renal clearance (Lu and Park, 2013). Longer circulation also allows the micelles to accumulate to a greater extent in areas with a defective or leaky vasculature such as tumors; via the enhanced permeability and retention (EPR) effect, which is the basis of passive targeting (Matsumura and Maeda, 1986; Maeda et al., 2000). Polymeric micelles possess a high structural stability due to the interactions between polymeric chains in the core-forming hydrophobic blocks, which allows them to retain encapsulated drugs and also be stable upon dilution in the body (Torchilin, 2002). Additional advantages of micelles include reduced side effects of the encapsulated drug, easy and reproducible scale-up, the ability to slow down opsonization and the possibility of longer circulation times when hydrophilic moieties such as PEG, that provide an effective steric barrier, are incorporated in the micelles. Finally, their surface can be modified with various ligands using different surface chemistries to produce targeted micelles (Torchilin, 2002; Sawant and Torchilin, 2010; Lu and Park, 2013).

### Passive targeting of polymeric micelles and modification for longevity

**Figure 2:**
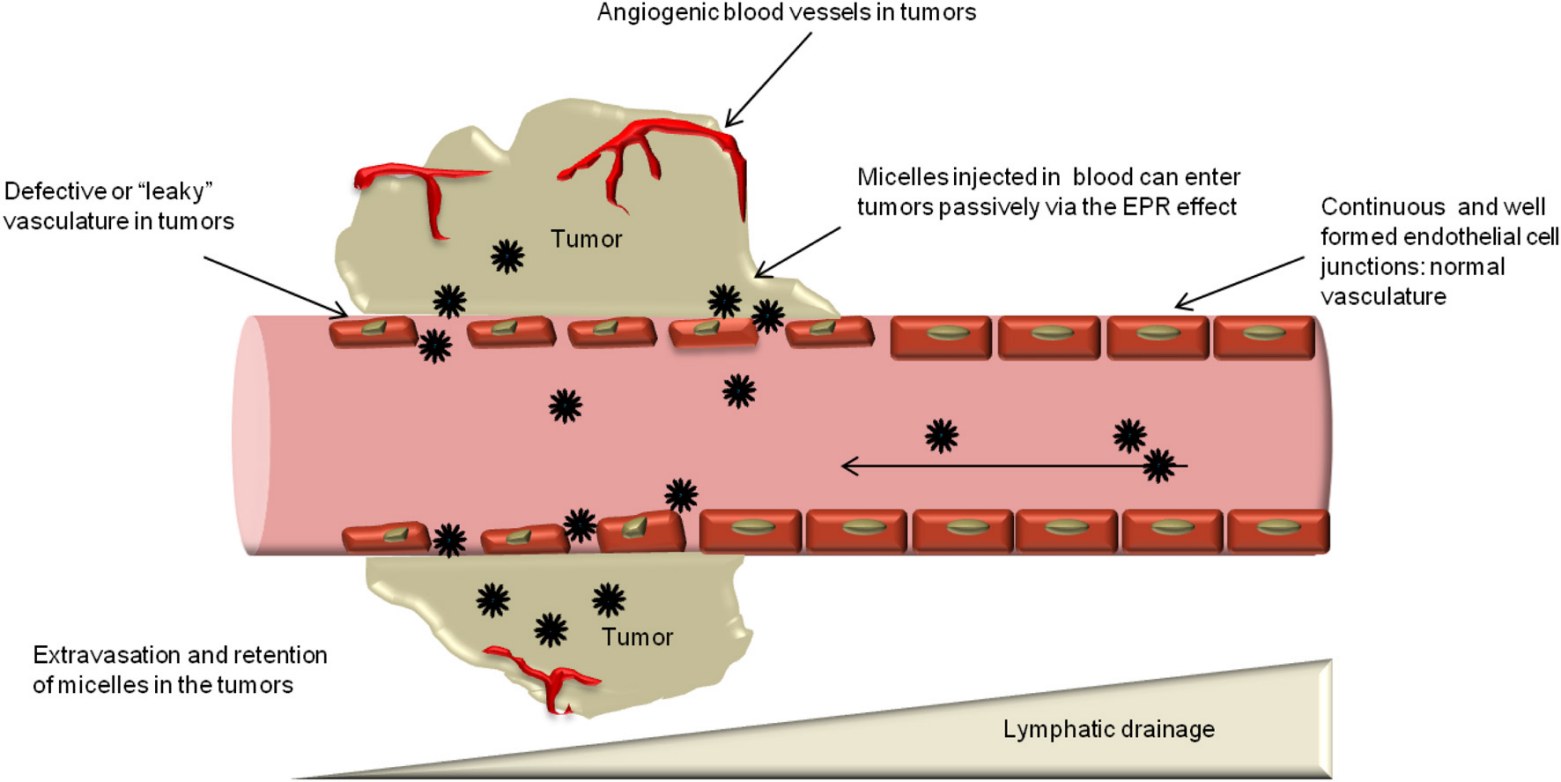
**Enhanced permeability and retention (EPR) effect and passive targeting**. Nanocarriers can extravasate into the tumors through the gaps between endothelial cells and accumulate there due to poor lymphatic drainage.

Passive targeting of nanocarriers including polymeric micelles relies on the tumor microenvironment. Their accumulation proceeds mainly via the EPR effect (Maeda et al., 2000). Tumor vasculature grows aberrantly to meet the ever-increasing nutrient and oxygen demand of the growing tumor, which leaves the endothelial cells poorly aligned with large fenestrations between them (Jain, 1987; Folkman, 1995; Roberts and Palade, 1997; Hobbs et al., 1998). This architectural abnormality and the production of vascular permeability factors like nitric oxide, bradykinin, matrix metalloproteinases (MMPs) and vascular endothelial growth factor (VEGF) make the tumor blood vessels highly permeable (Wu et al., 1998; Fang et al., 2011). The growing tumor cells also compress the lymph vessels, particularly in the central portion of the tumor, causing them to collapse, resulting in poor lymphatic drainage from tumors (Padera et al., 2004). Both these phenomena—the increased vascular permeability and the defective lymphatic drainage not only allow leakage of blood plasma components and nanoparticles (e.g., micelles) into tumor tissues, but also allow them to be retained there (Maeda et al., 2000; Iyer et al., 2006). This phenomenon is termed the EPR effect (Matsumura and Maeda, 1986) (Figure 2).

Although the EPR effect plays a crucial role in the accumulation of polymeric micelles, other factors such as the size and surface characteristics of micelles also determine the effectiveness of passive targeting (Torchilin, 2007; Ganta et al., 2008). Longevity of the nanocarrier in circulation is very important for passive targeting (Torchilin and Trubetskoy, 1995; Torchilin, 2006; Petros and Desimone, 2010). Generally, longevity is imparted to polymeric micelles by flexible, hydrophilic polymer coatings like PEG which can be grafted onto their surface (Torchilin and Trubetskoy, 1995; Torchilin, 2006). PEG acts as a steric barrier and is effective in preventing rapid opsonization of micelles by the MPS, lengthening their circulation time in the blood. This, in turn, gives micelles a better chance of extravasation through the leaky vasculature and slow accumulation in the tumor via the EPR effect (Mahmud et al., 2007; Torchilin, 2011). The steric barrier provided by PEG shields the surface charge of micelles, hinders their interaction with blood components and limits adsorption of plasma proteins on their surface (Torchilin, 2006).

The size of polymeric micelles also has a crucial role in the EPR effect-mediated accumulation as discussed in the previous section. Tumor vasculature cutoff sizes can vary between tumors (200–800 nm), and determine the diffusion and accumulation of molecules within the tumor interstitium (Yuan et al., 1995; Torchilin, 2011). The benefit of small sized polymeric micelles, which are well below the cutoff limit for most tumors, ensures that they remain in circulation for longer intervals without being taken up by MPS and eventually enter the tumor vasculature through the EPR effect. Some examples of polymeric micelle formulations exploiting the passive targeting effect are listed in Table 1.

**Table 1:** **Passively targeted therapeutic preparations of polymeric micelles**.

**Micelle components/formulation**	**Drug(s)**	**References**
**PRE-CLINICAL**		
PEG2000-PE	Docetaxel	Tong et al., 2012
PEG2000-PE/Vitamin E	Paclitaxel and curcumin	Abouzeid et al., 2014
	Paclitaxel and Elacridar	Sarisozen et al., 2012
PEG2000-PE/Hydrogenated phosphatidylcholine (PEG200-PE/HSPC)	Doxorubicin	Wei et al., 2012b
Adamantine terminated PEG and β-cyclodextrin based 7 armed poly(L-glutamic acid) (mPEG-Ad@β-CD-7PLGA/CDDP)	CDDP	Yong et al., 2013
Stearate grafted dextran	Doxorubicin	Du et al., 2010
mPEG-b-poly(D,L-lactide)	Docetaxel	Li et al., 2012d
Pluronic P123/F127	Paclitaxel	Zhang et al., 2010, 2011b
**CLINICAL**		
Genexol®-PM, mPEG-PDLLA (Ph-IV/approved in Korea)	Paclitaxel	Kim et al., 2004
NK105, PEG-p(Asp) (Ph-III)	Paclitaxel	Hamaguchi et al., 2007; Kato et al., 2012
SP1049C, Pluronic L61 and F127 (Ph-III)	Doxorubicin	Danson et al., 2004; Valle et al., 2011
NK012, PEG-P(Glu)-SN38 (Ph-II)	SN-38	Matsumura et al., 2004; Koizumi et al., 2006
NC-6004, PEG-P(Glu)-cisplatin (Ph-I/II)	Cisplatin	Plummer et al., 2011
NK911, PEG-P(Asp)-DOX (Ph-II)	Doxorubicin	Matsumura et al., 2004
NC-4016, PEG-P(Glu) DACHPt (Ph-I)	DACHPt^b^	Cabral et al., 2005, 2007

DACHPt, Dichloro-(1,2-diaminocyclohexane) platinum (II),

b CDDP, cis-dichlorodiamine platinum (II).

Although passive targeting is useful clinically, it is not without its drawbacks. Major impediments to passive targeting include the inherent tumor heterogeneity wherein cutoff sizes could vary between tumors, and even within the same tumor. The vasculature may exhibit varying porosities and consequently different permeabilities (Monsky et al., 1999; Prabhakar et al., 2013). The implicit diversity in tumors may lead to some areas not showing the characteristic EPR effect and some tumor vessels may not be “leaky” which leads to heterogeneous extravasation and delivery of drug vehicles (Yuan et al., 1995, 1996; Fang et al., 2011). In addition, nanocarriers such as polymeric micelles that are modified with a biocompatible surface coating of the hydrophilic PEG face the so called “PEG dilemma” since PEG may interfere with their endosomal escape and intracellular uptake into tumors (Hatakeyama et al., 2011). Also, only passive accumulation of micelles based on the EPR effect cannot ensure their effective delivery to molecular targets within tumors (Mahmud et al., 2007). It is thus clear that passive targeting alone may not suffice and additional approaches like active targeting which enable more selective and robust target recognition may need to be utilized (Torchilin, 2006; Peer et al., 2007).

### Active targeting of polymeric micelles

**Figure 3:**
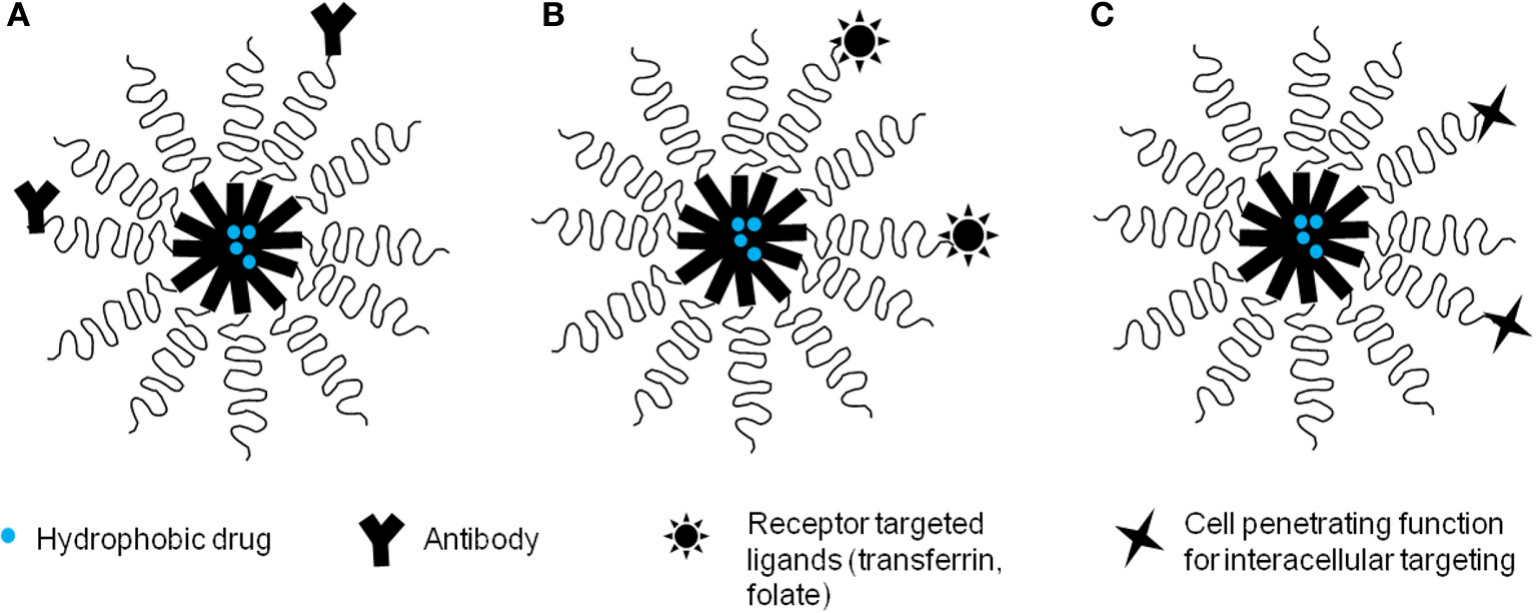
**Drug-loaded polymeric micelles with various targeting functions. (A)** Antibody-targeted micelles **(B)** ligand-targeted micelles **(C)** Micelles with cell-pentrating function.

Tumor cells and/or tumor vasculature frequently show increased expression of certain molecules (antigens or receptors) which are generally not expressed, or present at low levels on the surface of normal cells and surrounding normal tissues (Park et al., 2008; Kamaly et al., 2012). Active targeting exploits this feature of cancer cells to allow selective accumulation of anti-cancer therapeutics in the tumor tissue, tumor cells or intracellular organelles of the cell (Nie et al., 2007). Polymeric micelles can be functionalized for active targeting by chemically modifying their surface with targeting ligands that show a strong specificity for antigens or receptors over-expressed on cancer cells (Torchilin, 2007; Park et al., 2008) (Figure 3). Usually, the targeting ligands can be attached to the water-exposed free termini of hydrophilic blocks (PEG) of the micelles, so that they extend above the PEG brush and avoid steric hindrance when binding to their target receptors (Torchilin, 2001, 2007). Actively targeted polymeric micelles decrease side-effects of drugs by allowing preferential accumulation in diseased cells and also facilitate cellular uptake by receptor-mediated endocytosis (Park et al., 2008; Danhier et al., 2010). Active targeting especially benefits intracellular delivery of macromolecules like DNA, siRNA and proteins. The anti-tumoral efficacy of actively targeted delivery vehicles derives from their enhanced cellular internalization rather than just an increased tumor accumulation (Kirpotin et al., 2006).

A wide variety of ligands have been investigated to date for active targeting of polymeric micelles. Some of the commonly used targeting ligands include antibodies and their fragments (Jin et al., 2011a; Sawant et al., 2013a; Zhao et al., 2013), peptides (Gülçür et al., 2013; Miura et al., 2013; Chung et al., 2014), proteins (Fonge et al., 2012; Riehle et al., 2013; Sawant et al., 2013b), aptamers (Xu et al., 2013b), sugar moieties (Sun et al., 2011a; Yang et al., 2011; Yu et al., 2013b), and small molecules like folate (Qiu et al., 2013; Yang et al., 2013).

Antibodies have been the most popular targeting ligands for polymeric micelles to date because of the diversity of their targets and the specificity of their interaction (Torchilin, 2004). Over the years, antibody engineering technologies have enabled the development of murine, chimeric, humanized and human monoclonal antibodies (mAbs) as well as antibody fragments (e.g., Fab, scFv, diabodies, triabodies and single-domain antibodies) (Holliger and Hudson, 2005; Weiner, 2007). Poly(ethylene)glycol-phosphatidyl ethanolamine (PEG2000-PE) micelles loaded with doxorubicin (DOX) and targeted with the anti-nucleosome mAb 2C5, which is specific for cancer cells were shown to be effective in a DOX-resistant ovarian cancer cell spheroid model. The 2C5 targeted DOX micelles showed better uptake and penetration and also induced greater cell death in spheroids compared to free DOX and non-targeted DOX micelles (Perche et al., 2012). In another example, anti-Her2 antibody Fab fragment was conjugated to temperature sensitive block copolymer micelles made from poly(N-isopropylacrylmide-*co*-N,N'-dimethylacrylamide)_118_-*b*-poly(_D,L_-lactide)_71_ (Li et al., 2012b). The dual function of temperature sensitive and Her2 targeted immunomicelles showed significant *in vitro* toxicity and accumulation, high *in vivo* stability, greater intra-tumoral accumulation and significant tumor inhibition in a Her2 over-expressing mouse model of gastric cancer compared to various controls (Li et al., 2012b). Although very popular as targeting ligands, antibodies do face some challenges. These include their large size (~150 kDa) which results in limited ligand densities on micelles, potential immunogenicity which may lead to rapid clearance, stability considerations and engineering challenges during scale-up manufacturing (Goldenberg and Sharkey, 2012; Kamaly et al., 2012). Finally the success of immunomicelles largely depends on the target antigen being truly “tumor-specific” so as to avoid side-effects (Firer and Gellerman, 2012).

Proteins and peptides have also been used extensively as targeting ligands for polymeric micelles. The transferrin receptor (TfR) is over-expressed in many cancers and offers an attractive option for the development of transferrin-targeted nanocarriers (Singh, 1999). Polymeric micelles may be modified either with endogenous ligand transferrin (Tf) or antibodies against TfR (Torchilin, 2006). Sawant et al. showed that drug (R547)-loaded PEG2000-PE micelles modified with Tf showed a greater interaction with TfR over-expressing A2780 ovarian carcinoma cells *in vitro* at 48 h compared with free drug and non-modified micelles. Tf-targeted micelles also exhibited greater cytotoxicity *in vitro* and a significant tumor growth inhibition in mice vs. the drug-loaded, non-targeted micelles (Sawant et al., 2013b). Other protein ligands like tumor necrosis factor related apoptosis inducing ligand (TRAIL), which binds to death receptors up-regulated in cancer cells to induce apoptosis (Skidan et al., 2009; Lee et al., 2011a; Riehle et al., 2013) and epidermal growth factor (EGF) which targets the EGF receptors over-expressed in many cancers (Zeng et al., 2006; Fonge et al., 2012) are also utilized for modification of polymeric micelles for active targeting.

Peptides are used as targeting ligands due to their small size, lower immunogenicity compared to proteins, better stability *in vivo*, relative ease of conjugation to polymeric micelles and lower costs (Kamaly et al., 2012). The arginine-glycine-aspartic acid (RGD) tri-peptide which targets integrin receptors (α_v_β_3_, α_v_β_5_) has been widely investigated. Recently, Miura et al. reported polymeric micelles self-assembled from PEG-*b*-poly(_L_-glutamic acid) and (1,2-diaminocyclohexane)platinum(II) (DACHPt), the parent complex of oxaliplatin, targeted with cyclic RGD (cRGD) for delivery of anti-cancer drugs to glioblastoma (Miura et al., 2013). Intravital confocal laser scanning microscopy (IVCLSM) revealed that the cRGD micelles accumulated rapidly and had a higher permeability within the tumor parenchyma compared to the non-targeted micelles. The rapid internalization of these micelles also led to significant antitumor effects in an orthotopic mouse model of U87 MG human glioblastoma compared to the controls (Miura et al., 2013). PEG2000-PE micelles were modified with vasoactive intestinal peptide (VIP) to target VIP receptors over-expressed in breast cancer (Dagar et al., 2012; Gülçür et al., 2013). Other peptides like Lyp-1 (Cys-Gly-Asn-Lys-Arg-Thr-Arg-Gly-Cys) which targets the p32 receptors (p32/gC1qR) over-expressed on some tumor cells (Wang et al., 2012c), cell-penetrating peptides like *trans-activating* transcriptional transactivator (TAT) from HIV-1 (Kanazawa et al., 2012; Taki et al., 2012) and octreotide which targets somatostatin receptors have also been used to modify polymeric micelles (Xu et al., 2013a). Some groups have reported the development of peptides with affinities for molecular targets over-expressed on different types of tumor cells or tumor vasculature using the phage-display technology (Mori, 2004; Petrenko, 2008). Such peptides have been utilized successfully to modify polymeric micelles for cancer-specific targeting (Wang et al., 2010c; Qian et al., 2013).

Small molecules are particularly attractive as targeting ligands for polymeric micellar carriers due to inherent advantages like a small size, ability to attain higher ligand densities over antibodies, reproducible and scalable manufacturing, less immunogenicity when compared to macromolecules, ease of conjugation using simple chemical methods and the large diversity of such ligands (Kamaly et al., 2012). Folate receptors (FR) are over-expressed in a number of cancers, and hence folate is widely used as a targeting ligand for cell-specific delivery in these cancers (Leamon and Low, 1991; Sudimack and Lee, 2000). DOX-loaded and folate-targeted poly(2-ethyl-2-oxazoline)-*b*-PCL micelles (FA-PEOz-PCL) showed better cellular uptake and exhibited lower IC_50_ values in FR over-expressing cells compared to non-targeted micelles. *In vivo*, they exhibited better anti-tumor efficacy and reduced toxicity compared to free DOX (Qiu et al., 2013). Other small molecules like biotin (Lin et al., 2013), galactose (Zhong et al., 2013), and mannose (Freichels et al., 2012) have also been reported for surface modification of polymeric micelles.

Aptamers—oligonucleotides which have the ability to fold into defined 3D structures and bind with high affinity and specificity to their target molecules (proteins, peptides or small molecules) are also gaining momentum as targeting ligands (Zhang et al., 2011c). A10 aptamer (Apt), which recognizes the extracellular domain of the prostate-specific membrane antigen (PSMA), was conjugated to unimolecular DOX loaded micelles consisting of a H40 dendritic core, an inner shell of hydrophobic PLA and an outer hydrophilic PEG shell (H40-PLA-PEG-Apt) for targeted prostate cancer therapy(Xu et al., 2013b). The Apt-targeted micelles exhibited a significantly higher uptake and hence cytotoxicity over the non-targeted micelles. *In vivo*, they also resulted in a much higher DOX uptake in tumors than non-targeted micelles or free DOX (Xu et al., 2013b).

Table 2 lists some examples of actively targeted polymeric micelles.

**Table 2:** **Some examples of actively targeted micelles**.

**Micelle components**	**Therapeutic agent**	**Targeting ligand**	**Target**	**References**
Poly(D,L-lactic-co-glycolic acid)-PEG	Doxorubicin	HAb18(Fab')_2_	Hepatocellular carcinoma cells	Jin et al., 2011a
PEG2000-PE	Curcumin and Doxorubicin	Anti-GLUT1 antibody	GLUT1 receptors	Abouzeid et al., 2013
	R547 (cyclin-dependent kinase CDK-inhibitor)	2C5 and Transferrin	Cancer cell surface associated nucleosomes and transferrin receptors	Sawant et al., 2013a
	DM-PIT1 analogs	TRAIL	Death receptors on cancer cells	Riehle et al., 2013
Poly(L-glutamic acid)-*g*-α-tocopherol/PEG	Docetaxel and Cisplatin	Cyclic(RGD)fk	α_*v*_β_3_ integrins	Song et al., 2014
DSPE-PEG2000	Curcumin	Vasoactive intestinal peptide (VIP)	VIP receptors	Gülçür et al., 2013
PEO-PPO-PEO	Epirubicin	Biotin	Biotin receptors	Lin et al., 2013
Poly(lactic acid)-PEG	Docetaxel	Folic acid	Folate receptors	Hami et al., 2014
Cholesterol modified glycol chitosan (CHGC)	Doxorubicin	Galactose	Asialoglycoprotein receptors	Yu et al., 2014
Azide (N3)-PEG-poly(ε-caprolactone)	TGX-221 (PI3K inhibitor)	PSMA a10 aptamer	Prostate specific membrane antigen	Zhao et al., 2012

DM-PIT-1, N-[(2-hydroxy-5-nitrophenyl)amino]carbonothioyl-3,5-dimethylbenzamide; PEO- PPO- PEO, poly(ethylene oxide)-co-poly(propylene oxide)-co-(polyethylene oxide).

### Stimuli-responsive polymeric micelles

An added sophistication to selective delivery of polymeric micelles can be brought about by utilizing certain cues inherently characteristic of the tumor microenvironment (intrinsic) or by applying certain stimuli to this region from outside the body (extrinsic) (Torchilin, 2009). Polymeric micelles can be engineered so as to respond to various intrinsic or extrinsic stimuli of physical, chemical or biochemical origins to achieve spatial and temporal control over the release of therapeutic payloads (Cheng et al., 2013). “Environmentally-responsive” or “smart” polymeric micelles can release their therapeutic payloads by undergoing structural modifications in response to the stimulus. The response may result in disintegration/destabilization, isomerization, polymerization or supramolecular aggregation of micelles (Fleige et al., 2012). The commonly encountered intrinsic stimuli in tumors include low pH, redox status of the cell, and the presence of certain over-expressed enzymes while the extrinsic stimuli include magnetic fields, light (UV, infrared or visible) and ultrasound. Hyperthermia is a stimulus that could be either intrinsic or extrinsic—intrinsically from inflammation, or extrinsically upon application of ultrasound or alternating magnetic fields in conjunction with magnetic nanoparticles which release heat (Torchilin, 2009).

The acidic pH in tumors results from extensive hypoxia and cell death which leads to production and accumulation of lactic acid (Tannock and Rotin, 1989). The pH in tumors is ~6.5 compared to ~7.4 in the normal tissues and drops even further in the intracellular organelles like endosomes (~5–6) and lysosomes (~4–5) (Gerweck and Seetharaman, 1996; Casey et al., 2010). These pH-gradients have been exploited successfully to design pH-sensitive polymeric micelles which can release their therapeutic payloads when they encounter a change in the pH of their microenvironment. A number of strategies have been explored to design pH-sensitive micelles, which have been reviewed elsewhere (Felber et al., 2012; Liu and Zhang, 2012; Chen et al., 2013; Li et al., 2013d).

The redox potential in cancer cells is elevated (100–1000-fold higher) due to the high intracellular concentration (2–10 mM) of glutathione tripeptide (γ-glutamyl-cysteinyl-glycine) (GSH) compared to its concentration (2–10 μM) outside cells (Saito et al., 2003). Moreover, tumor cells also show elevated GSH levels compared to normal cells. Polymeric micelles with disulfide bonds have been designed to hold the cargo (drugs, siRNA, DNA or proteins) under normal conditions and release it upon destabilization in the reducing conditions found inside cancer cells that can convert disulfide linkages to thiols (Torchilin, 2009; Wei et al., 2012a). The disulfide linkages can be incorporated in the hydrophobic backbone (Li et al., 2013e), at the junction of hydrophobic and hydrophilic blocks (Li et al., 2012a), or by incorporating reduction sensitive cross-links in the micelle core (Li et al., 2011), shell (Koo et al., 2012) or the core-shell interface (Hossain et al., 2010). siRNA has also been delivered effectively using redox-sensitive micelles (Matsumoto et al., 2009; Musacchio et al., 2010). Gradients of oxygen tension within the tumors can be exploited to design hypoxia sensitive nanocarriers. In one of the first studies of its kind, Perche, Biswas et al. reported a hypoxia activated nanocarrier for siRNA to achieve the down-regulation of a model gene (GFP) *in vitro* and *in vivo* (Perche et al., 2014). Here, azobenzene was used as a hypoxia-responsive, bio-reductive linker for hypoxia-targeted delivery of siRNA from PEGylated nanopreparations upon PEG cleavage. The nanocarrier consisted of PEG2000, azobenzene, polyethyleneimine (PEI 1.8 kDa) and 1,2-dioleyl-sn-glycero-3-phosphoethanolamine (DOPE) units, known as PAPD. The azobenzene was linked to PEG2000 (for stability in circulation) at one end and to a PEI-DOPE conjugate (to complex siRNA) at the other end to form the nanocarrier. PAPD showed a hypoxia-dependent cellular uptake and accumulation and resulted in 30-40% GFP down-regulation in a number of GFP expressing cell lines under hypoxic conditions. In A2780/GFP tumor bearing mice, GFP down-regulation was detected by *ex-vivo* imaging (24%) and flow cytometry (32%) after intravenous (i.v.) administration of PAPD/siRNA which correlated well with the *in vitro* results (Perche et al., 2014).

Enzyme-sensitive micelles take advantage of the altered expression profile of certain enzymes in cancer or other diseases to deliver therapeutics to the desired targets (Mura et al., 2013). Enzyme sensitive moieties can be used to modify the polymers (main chain or side groups), which upon recognition by the enzyme cause structural changes in the micelles. Alternatively, these moieties may also be recognized by a product of the enzymatic reaction. Another option is to modify the micelle surface with peptides or oligonucleotides that can cause physical changes in the micelles upon enzymatic transformation (De La Rica et al., 2012; Hu et al., 2012). The enzymes most frequently dysregulated in cancer include hydrolases (proteases, lipases and glycosidases), metabolic enzymes including those involved in glycolysis and fatty acid synthesis and oxidoreductases (De La Rica et al., 2012). The matrix metalloproteinase (MMP) family of enzymes (MMP-2 and 9 in particular) is primarily linked to cancer progression and metastasis. To that end, polymeric micelles containing MMP-sensitive linkers have been reported for tumor-specific delivery of drugs and siRNA in response to the over-expressed MMPs (Li et al., 2013b; Zhu et al., 2014). DOX-loaded polysaccharide-lecithin reverse micelles, with a triglyceride outer shell that was sensitive to hydrolysis by lysosomal acid lipase, were reported to overcome multi-drug resistance (Su et al., 2013). Enzyme-responsive polyion complex micelles (PICs) that disintegrate in response to phosphatase and acetylcholinesterase respectively have been developed by Zhang and co-workers (Wang et al., 2010a; Xing et al., 2012).

Among the extrinsic stimuli, ultrasound has been investigated widely as a trigger for drug release from polymeric micelles (Husseini and Pitt, 2008b; Husseini et al., 2009; Mohan and Rapoport, 2010; Rapoport et al., 2010). Ultrasound refers to the application of pressure waves above a frequency of 20 kHz to spatially and temporally control drug release (Husseini and Pitt, 2008a). Pluronic® micelles have been investigated extensively for ultrasound-triggered delivery of both drugs as well as nucleic acids (Chen et al., 2006; Husseini et al., 2013). While low frequency ultrasound (20–100 kHz) can penetrate deeper into the body tissues than high frequency ultrasound (1–3 MHz), it cannot be focused as well (Rapoport, 2012). *In vitro*, ultrasound can perturb the micelle structure and cause the release of therapeutic payloads due to cavitation. *In vivo*, this mechanical effect of ultrasound may also be accompanied by local hyperthermia, which could lead to increased micelle extravasation and accumulation in the tumor tissues (Rapoport, 2012). Ultrasound-sensitive, paclitaxel (PTX)-loaded block copolymer micelles of methoxy PEG and poly(D,L-lactide) (MePEG-b-PDLLA) resulted in increased PTX accumulation and subsequently increased cytotoxicity in both drug-sensitive and drug-resistant (P-glycoprotein expressing) cell lines (Wan et al., 2012).

Magnetic field has also been explored as an extrinsic stimulus for polymeric micelles. Micelles can be loaded with drugs as well as superparamagnetic iron oxide nanoparticles (SPIONS) like magnetite (Fe_3_O_4_) or maghemite (Fe_2_O_3_) which allows them to be manipulated under the guidance of an externally applied permanent magnet or an alternating magnetic field to control either the drug release, result in a temperature increase or even both when used alternately (Torchilin, 2009; Mura et al., 2013). Wang et al. reported SPION-loaded poly (D,L-lactide)-*b*-mPEG (mPEG-PLA) micelles coated with chitosan and PEI (CP-mag-micelles) for delivery of plasmid DNA and magnetic resonace imaging (MRI). The CP-mag micelles had high MRI relaxivity, showed significantly higher transfection efficiencies compared to PEI or lipofectamine and a single injection of plasmid-bearing CP-mag micelles could express genes *in vivo* for at least 1 week (Wang et al., 2012b). In another study, folate-targeted, DOX-loaded magnetic nanomicelles made from Pluronic F127 and PLA showed enhanced accumulation *in vitro* and *in vivo* in the presence of an external magnetic field (Huang et al., 2012). Magnetic heating was utilized to trigger drug release from PEG-*b*-PCL micelles loaded with iron oxide nanoparticles and DOX. The release of DOX was faster when the micelles were heated above the melting point of their PCL cores (Glover et al., 2013).

Temperature is one of the most widely investigated stimuli for drug delivery and has been extensively explored for cancer treatment. Thermo-responsive micelles are constructed from thermo-sensitive blocks which can undergo a sharp change in phase that destabilizes the micelles and triggers the release of the drug (Torchilin, 2009; Mura et al., 2013). The most widely used polymer for such micelles is poly(N-isopropyl acrylamide) (PNIPAAm), which exhibits a lower critical solution temperature (LCST) of 32°C. It undergoes a phase transition from hydrophilic (coil) to hydrophobic (globule) state above its LCST. The LCST can be varied by controlling the hydrophilic and hydrophobic polymer composition (Dimitrov et al., 2007; Kang et al., 2008). Yang et al. reported camptothecin (CPT)-loaded micelles formed from comb-like copolymers of mPEG blocks and hydrophobic polyacrylate (PA) backbones, with thermosensitive PNIPAAm graft chains (mPEG-*b*-PA-*g*-PNIPAAm). The micelles showed a thermo-responsive hydrophilic to hydrophobic phase transition with a LCST from 40 to 45°C. CPT release from the micelles was continuous, without an initial burst release, and was accelerated above the LCST. The CPT- loaded thermo-responsive micelles were selectively cytotoxic to cancer cells while avoiding toxicity to normal cells, unlike the free drug (Yang et al., 2014). Prabharan et al. reported thermo-sensitive poly (*N*-vinylcaprolactam)-*b*-PEG micelles loaded with 5-fluorouracil (5-FU) and coupled with folate as a targeting agent to target FR over-expressing cancer cells and showed controlled drug release at 37°C (Prabaharan et al., 2009).

Light-sensitive micelles can utilize ultraviolet (UV), visible or near infrared (NIR) light to trigger drug release with excellent remote spatio-temporal control (Mura et al., 2013). Some recent reviews have discussed the design principles of such photo-responsive micelles and mechanisms of photo-induced drug release from delivery carriers (Schumers et al., 2010; Fomina et al., 2012; Gohy and Zhao, 2013). Photo-responsive groups can be incorporated within the micelle core, corona or at the core-shell interface in the design of light-sensitive micelles (Gohy and Zhao, 2013). Generally, the chromophores or light-sensitive moieties are incorporated within the core, or conjugated to it (Oerlemans et al., 2010). Azobenzenes and their derivatives comprise the most commonly encountered reversible photo-responsive groups. They undergo a reversible *trans-cis* photoisomerization upon UV light irradiation which converts the apolar *trans* isomer to a polar *cis* isomer, while visible light reverses this isomerization (Zhao, 2007). To develop photo-sensitive micelles with increased dispersion stability, Boissiere et al. reported flower-like micelles containing hydrophobically modified PNIPAAm with multiple azo-benzene segments incorporated into the main chain (Boissiere et al., 2011). The micelles responded to both UV and visible light by undergoing reversible *trans-cis* isomerization and remained well dispersed even above the LCST of PNIPAAm due to the multiple chain-folding of multi-azo-PNIPAAm chains caused by aggregation of azobenzene moieties (Boissiere et al., 2011). Recently, spiropyran- initiated hyperbranched polyglycerol (SP-*hb*-PG) micelles were reported, which responded to UV/visible light and could dissociate due to conversion of the hydrophobic chromophore SP to zwitterionic and hydrophilic merocyanine (ME) (Son et al., 2014). Chromophores like coumarin, o-nitrobenzyl, stilbene, dithienylethene and 2-diazo-1,2-napthoquinone (DNQ) have also been employed in light-responsive micelles, which can respond either to UV/visible or NIR irradiation to undergo structural or phase changes and trigger the drug release from micelles (Chen et al., 2011; Jin et al., 2011b; Menon et al., 2011; Liu et al., 2012; Cao et al., 2013). Table 3 highlights selected examples of stimuli-responsive polymeric micelles from the recent literature.

**Table 3:** **Some examples of stimuli-responsive polymeric micelles**.

**Micelle components**	**Payload**	**Stimulus**	**Mechanism**	**References**
**INTERNAL STIMULI**				
Chondroitin sulfate-histamine (CS-His)	Doxorubicin	pH	Protonation of His residue alters the hydrophilic-hydrophobic balance of CS-His conjugate to release DOX at low pH	Yu et al., 2013a
Poly(ketal adipate)-co-PEG (PKA-PEG)	Camptothecin/Nile red	pH	Ketal linkages in the backbone cleaved under acidic pH to release payloads	Lee et al., 2013
mPEG–PCL–CH_2_R_4_H_2_C (cell penetrating peptide) (C: Cys, H:His, R:Arg)	VEGF siRNA	GSH (redox)	siRNA condensed in micelles through disulfide links via Cys (in CPP) released upon S-S cleavage in cytoplasm.	Tanaka et al., 2013
siRNA-SS-Poly(D,L-lactic co-glycolic acid)/linear PEI	GFP siRNA	GSH (redox)	Reductive cleavage of disulfide bond to release GFP siRNA in cytoplasm	Lee et al., 2011b
PEG-*b*-poly(2-methyl-2-carboxyl-propylene carbonate)-*g*-Gemcitabine-*g*-dodecanol (PEG-*b*-PCC-*g*-GC*-g*-DC)	Gemcitabine	Enzyme Cathepsin B	Cathepsin B cleaves amide bonds used to conjugate drug to polymer and enhances release, or acts on amide bonds in hydrolytically dissociated micelles to release free drug	Chitkara et al., 2013
PEG-b-poly(L-glutamic acid)-*b*-poly(L-phenylalanine) (PEG-*b*-PGlu-*b*-PPhe)	Cisplatin (CDDP) and paclitaxel	Enzyme Cathepsin B	Cathepsin B induced disintegration of polypeptide based building blocks in micelles to release drugs, also facilitated by pH	Desale et al., 2013
**EXTERNAL STIMULI**				
Pluronic F105 (PEO-PPO-PEO)	Doxorubicin	Ultrasound	70 kHz ultrasound induced transient cavitation led to micelle disruption to release DOX	Husseini et al., 2013
Hetero-assembly of mPEG-b-P(L-lysine) micelles with siRNA and gas cored liposomes to form siRNA nanobubbles (NB)	SIRT2 siRNA	Ultrasound	Low freq. ultrasound induced cavitation of siRNA-nanobubbles (NB) to release siRNA-micelles from NB and deliver them to the cell cytoplasm by a sonoporation effect	Yin et al., 2013
Folic acid/dextran-retinoic acid	Doxorubicin and magnetic NPs	Magnetic field	Localization and internalization of micelles in cells driven by MNPs in response to external magnetic field (0.42T)	Varshosaz et al., 2013
PEG-*b*-PCL	Doxorubicin and SPIO	Magnetic field	Hyperthermia due to heating of SPIO caused DOX release from micelles	Glover et al., 2013
P-(NIPAAm-co-NHMAAm)-b-PCL	Doxorubicin	Thermo-responsive	Increased DOX release above LCST (38°C) due to hydrophilic to the hydrophobic transition in the poly-(NIPAAm-co-HMAAm) shell resulting in collapse of micelle sturcture	Wang et al., 2014
PEC micelles assembled from chitosan-g-PNIPAAm and carboxymethyl cellulose-g-PNIPAAm	5-fluorouracil	Thermo-responsive (also pH responsive)	Deformation of micelles and controlled release of 5-FU above LCST (37°C)	Li et al., 2013a
PEO-*b*-P(LGA-*co*-COU)	Paclitaxel/Rifampicin	NIR Light	Two-photon absorption of NIR light by coumarin moiety causes shift in the hydrophilic-hydrophobic balance toward destabilization of micelles to release drugs	Kumar et al., 2012
Dialkoxycyanostilbene polymethacrylate-*b*-PEO (PDACS-b-PEO)	Curcumin	UV Light	Trans-cis photoisomerization of stilbene upon UV irradiation reduces hydrophobicity of polymer and disrupts micelles to release curcumin	Menon et al., 2011

SPIO, Superparamagnetic iron oxide; P-(NIPAAm-co-NHMAAm)-b-PCL, P-(N,N-isopropylacrylamide-co-N-hydroxymethylacrylamide)-b-caprolactone; PEC, Polyelectrolyte complex micelles; 6-bromo-7-hydroxycoumarin-4-ylmethyl (COU); NP, Nanoparticles.

## Multifunctional polymeric micelles

### Defining the concept of multifunctionality

**Figure 4:**
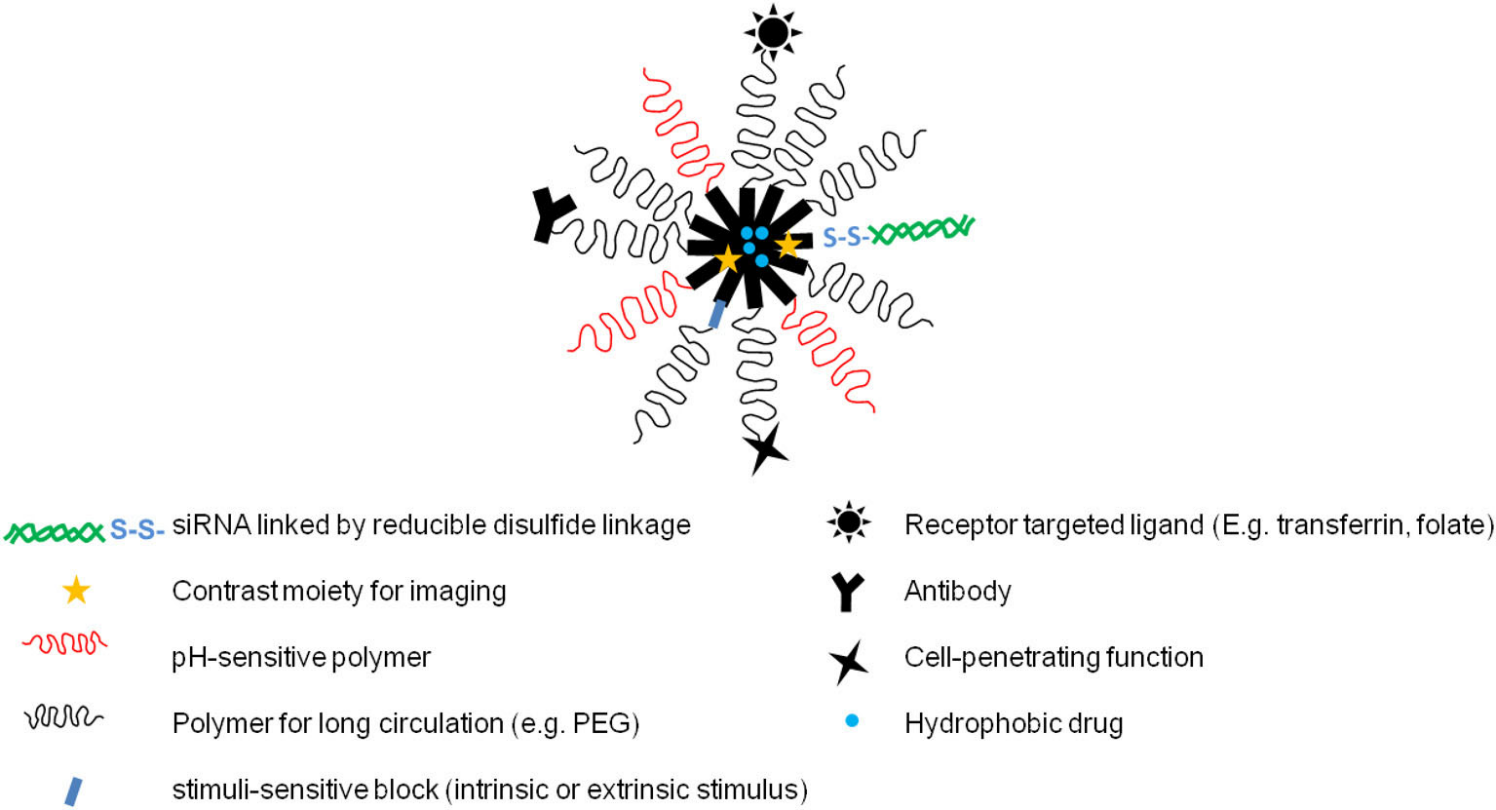
**A hypothetical multifunctional polymeric micelle**. Multifunctional polymeric micelles can be designed to incorporate two or more of these different functions.

The preceding sections describe various targeting strategies and modifications of polymeric micelles including very basic modifications for longevity which are important for passive targeting of therapeutics solubilized within micelles, surface modification with ligands to allow for selective targeting as well as intracellular delivery of drugs and nucleic acids and finally the modifications which allow micelles to respond to a number of intrinsic and extrinsic stimuli for “triggered” drug release at disease sites. Polymeric micelles, while allowing for the different modifications individually, also offer a platform that allows for integration of multiple components within a single micelle. We can thus engineer micelles to have two or more different modifications that enable them to simultaneously or sequentially perform important therapeutic and diagnostic functions (Torchilin, 2006). Such micelles which can combine a number of distinct functions or properties within a single carrier, with each individual component functioning seamlessly and in perfect coordination with the other components, give rise to the so-called “multifunctional” micelles. In addition to the various modifications just mentioned, contrast or reporter moieties can be incorporated within micelles which allow real-time imaging of these micelles and their accumulation within cells (Torchilin, 2006). Thus, an ideal multi-functional micelle may simultaneously deliver drugs/biologics, circulate in the body for extended periods, allow for passive or active targeting-mediated accumulation, respond to various stimuli to release incorporated cargoes, and may also provide diagnostic and therapeutic monitoring abilities (Figure 4). The key to developing such multifunctional micelles is to ensure that each of the components function in a coordinated manner so that the combined contribution of each adds up to something better than the individual components themselves. It is analogous to an orchestra, where each instrument must be played in perfect harmony to create a beautiful symphony. Cancer is a complex disease characterized by molecular and phenotypic heterogeneity within and between tumor types which makes chemotherapy very challenging. Although, molecularly targeted therapies have been developed, a selection of tumor cells may still escape the targeted pathway and lead to adaptive resistance, causing the failure of therapy (Blanco et al., 2009). It follows from the discussion above that using a multi-faceted approach for targeting cancer seems imperative. While it may be difficult to incorporate all the features of the “ideal” multifunctional polymeric micelle, a combination of two or more of the desirable features is feasible and also necessary for the success of cancer therapy using micellar nanocarriers. A lot of the focus of current research has been in the direction of such multifunctional micelles for their obvious advantages in enhancing the efficacy, maximizing the safety and specificity of existing as well as novel chemotherapy regimens.

### Multifunctional micelles for delivery of drugs

Multifunctional polymeric micelles have been investigated extensively in recent times for delivery of drugs as well as nucleic acids. A number of interesting combinations have been explored for drug-loaded multifunctional polymeric micelles, a select few of which are discussed here.

Multifunctional micelles made of block copolymers of PLGA-PEG were reported for the combined delivery of DOX and PTX (Duong and Yung, 2013). A cell penetrating moiety (TAT) and a targeting ligand (folate) were used to modify PLGA-PEG to achieve an enhanced therapeutic effect for the drug combination vs. the single drugs. The authors tested single and dual drug-loaded micelles modified either with folate or with both TAT and folate ligands respectively, and found that dual drug-loaded micelles modified with both ligands exhibited a significantly lower IC_50_ value in KB cells (mouth epidermal carcinoma) compared to the single drug-loaded micelles. Although a synergistic effect was observed with both methods (co-delivery of two single drug-loaded micelles and dual drug-loaded micelles), the authors hypothesized that the drug ratio would be better maintained in the dual drug-loaded multi-functional formulation *in vivo* compared to the co-delivery of dual targeted, single drug-loaded micelles (Duong and Yung, 2013). MRI-responsive micelles typically incorporate magnetic nanoparticles within the core, but in a novel approach Li et al. developed hybrid micelles fabricated from Pluronic F127 and a peptide-amphiphile (PA) consisting of segments of a palmitic part, aspartic acid residue and three histidine residues (pal-AAAAHHHD), in which the magnetic nanoparticles were embedded in the shell (Li et al., 2013f). Micelle formation was driven by hydrophobic interactions between hydrophobic segements of Pluronic F127 and PA, with hydrophobic DOX encapsulated within the micelles. The chelating ability of the aspartic acid and histidine residues in the peptide enabled the *in situ* growth of magnetic nanoparticles within the shell by chemical precipitation of iron oxides. The shell-embedded magnetic nanoparticles significantly improved stability and retarded the release of DOX from the hybrid micelles, due possibly to their crosslinking effect on the shell. The DOX-loaded hybrid micelles also served as effective T2 weighted MRI contrast agents both *in vitro* and *in vivo* and also had the advantage of a simple, convenient and “green” synthesis under ambient conditions devoid of organic solvents (Li et al., 2013f).

**Figure 5:**
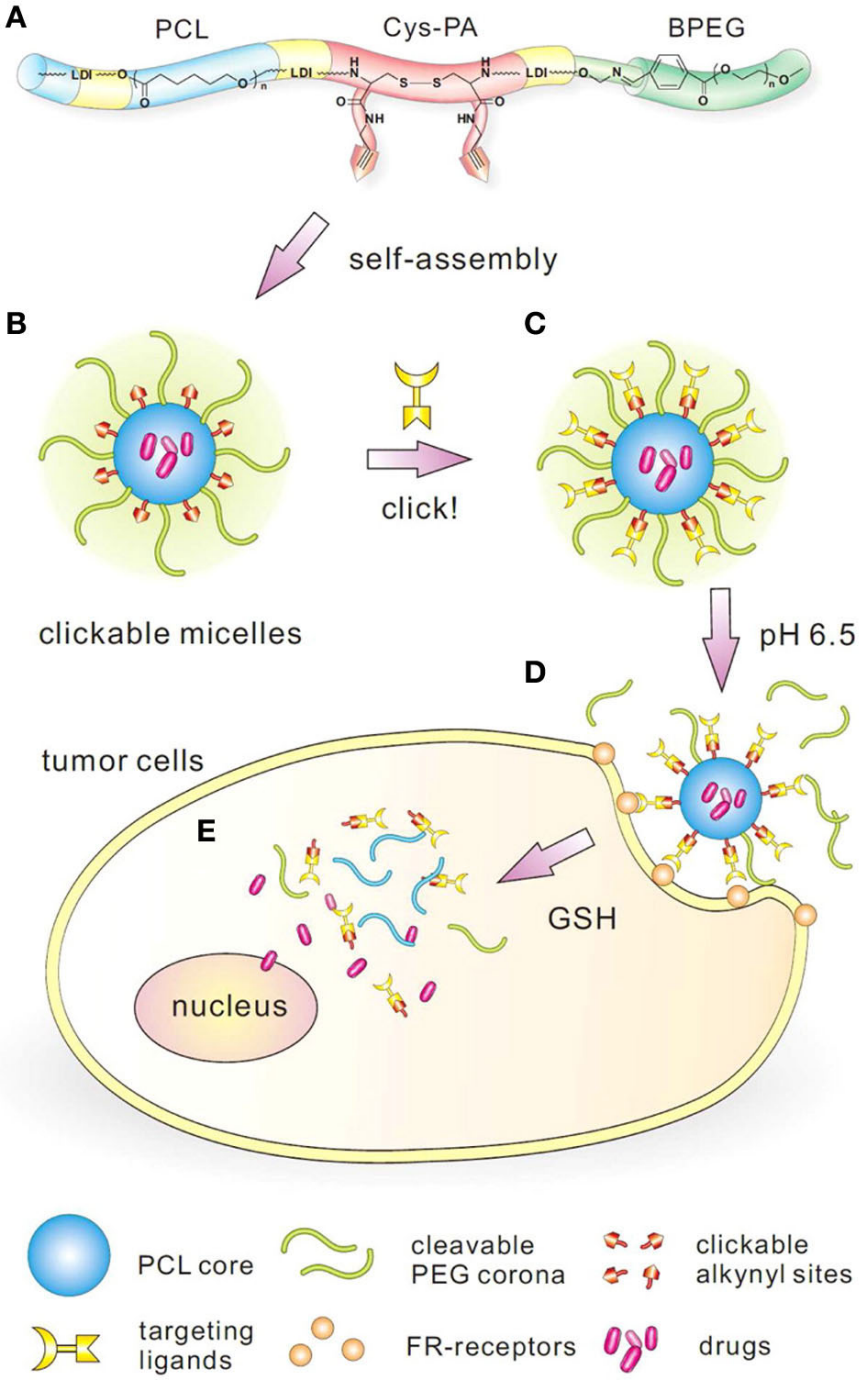
**Design and construction of targeting-clickable and tumor-cleavable polyurethane nanomicelles. (A)** Schematic molecular structure of multiblock polyurethanes; **(B)** self-assembled clickable polyurethane nanomicelles; **(C)** conjugation of folate ligand via click chemistry; **(D)** extracellular pH-activated detachment of PEG shell through the cleavage of benzoic-imine linkage; **(E)** intracellular drug release triggered by the cleavage of disulfide bond in response to GSH. Reprinted with permission from Song et al. (2013). Copyright © 2013 American Chemical Society.

Another novel, targeting-clickable and tumor-cleavable multi-block polyurethane (MPU) nanomicelle formulation was reported for the multifunctional delivery of chemotherapeutics (Song et al., 2013). The polyurethane backbone was composed of poly(ε-caprolactone) (PCL) and L-lysine ethyl ester diisocyanate (LDI). It was extended using a L-cystine-derivatized chain extender bearing redox-sensitive disulfide bonds in the backbone and two clickable alkynyl groups (Cys-PA) in the side-chain for post-functionalization with ligands (Figure 5).

The terminal group was comprised of a detachable mPEG with a pH-sensitive benzoic-imine linkage (BPEG) which could cleave in the slightly acidic conditions (pH, 6.5–7.2) found in the extracellular tumor environment. The prepared multi-block polyurethanes self-assembled into nanomicelles in an aqueous solution, and DOX was loaded into micelles using the dialysis method. Folic acid (FA) was modified with azide and conjugated to the micelles by the copper catalyzed alkyne-azide cycloaddition (CuAAC) click reaction. The MPU micelles were sensitive to multiple stimuli (intracellular GSH-sensitive drug release and extracellular pH-sensitive PEG detachment) and had a high DOX loading capacity. The folate-targeted micelles showed a higher cellular uptake and increased cytotoxicity over non-targeted micelles in FR positive HeLa cells *in vitro* (Song et al., 2013). The same group also reported multifunctional MPU micelles loaded with PTX and targeted with C225 monoclonal antibody against the EGFR extracellular domain for intracellular drug delivery. These micelles possessed hydrazone linkages for pH-responsivity, cell penetrating gemini quaternary ammonium (GQA) cationic groups for enhancing cell internalization and a tripeptide containing reactive carboxyl anion groups to provide an active site for conjugation of the targeting antibodies (Ding et al., 2013).

**Figure 6:**
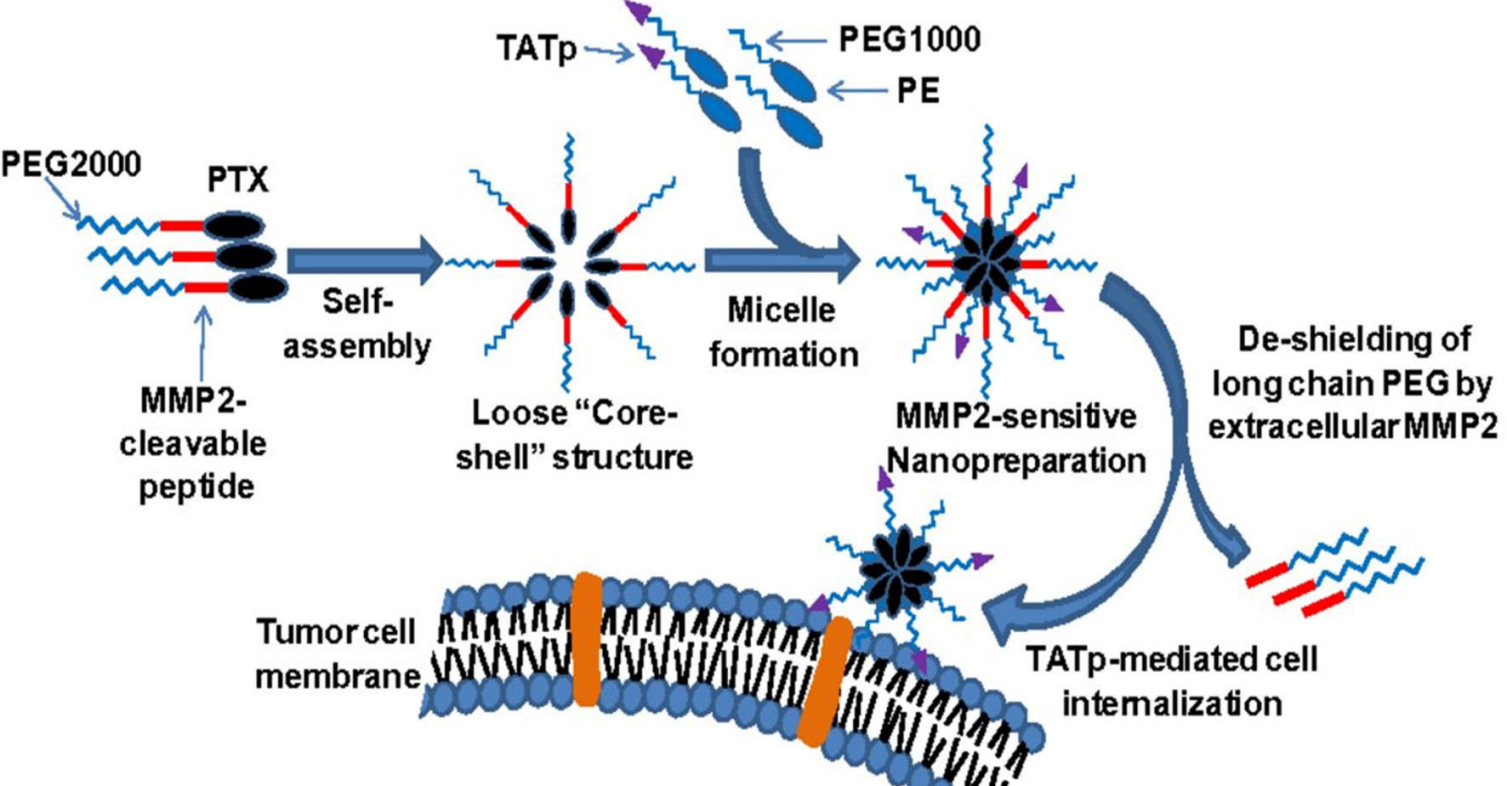
**MMP-2 sensitive nanopreparations**. Modified from Zhu et al. (2013). Copyright © 2013 PNAS.

Zhu et al. developed a unique tumor-targeted micellar drug-delivery platform combining several key strategies in a collaborative manner to simultaneously counter many of the key challenges faced by chemotherapeutics (Zhu et al., 2013). The authors synthesized a self-assembling drug-polymer conjugate/prodrug, PEG2000-peptide-PTX with an MMP cleavable linker GPLGIAGQ between PEG and PTX. This functioned as a tumor environment-sensitive water-soluble PTX prodrug and an MMP2-sensitive building block for a PTX nanopreparation. MMP-2 sensitive micelles were composed of the PTX prodrug PEG2000-peptide-PTX (50 mol%) and two other polymers, TATp-PEG1000-PE (10 mol%) (cell-penetration enhancer) and PEG1000-PE (40 mol%) (nanocarrier building block) following their self-assembly in an aqueous environment as shown in Figure 6. In the MMP-2 sensitive nanopreparation (TATp-PEG1000-PE/PEG2000-peptide-PTX), PTX was loaded in the core surrounded by the hydrophilic PEG shell. After accumulation of micelles within tumors by the EPR effect, the peptide linker was cleaved by the upregulated extracellular MMP2, to allow liberation of the active PTX and exposure of the hidden TATp (attached to a shorter PEG chain) for cell internalization. The MMP2-sensitive, TATp-modified, micellar formulation showed a high PTX loading (15% w/w) with low risk of premature drug release/leakage, had a superior cell internalization and hence cytotoxicity *in vitro*, as well as greater tumor targeting and anti-tumor efficacy *in vivo* compared to the non-MMP2 sensitive formulation, free PTX or conventional micelles (PEG2000-PE/PTX). This formulation thus exhibited its potential for cancer cell-selective intracellular delivery for enhanced cancer therapy by combining multiple delivery strategies including self-assembly, PEGylation, accumulation by EPR effect, stimulus sensitivity, cell-penetrating moiety and the concept of a prodrug (Zhu et al., 2013).

In addition to micelles made from common diblock copolymers, asymmetric tri-block copolymers have also been reported. Bastakoti et al. developed multifunctional core-shell-corona polymeric micelles using a special type of asymmetric tri-block copolymer poly(styrene-acrylic acid-ethylene glycol) (PS-PAA-PEG) (Bastakoti et al., 2013). The self assembly of PS-PAA-PEG in aqueous solutions produces micelles with a PS core, an anionic PAA shell and a neutral and hydrophilic PEG corona. Nile red (NR), a phenoxazine dye was loaded in the PS core using the dialysis method. Cisplatin was incorporated in the pH-sensitive PAA shell to allow for faster release at mildly acidic pH (5.0) compared to the normal physiological pH. The interaction of cisplatin with micelles was enhanced due to the abundant carboxylic groups on the PAA shell. Calcium phosphate (CaP) was selectively mineralized on the PAA shell, which enhanced the fluorescence intensity of NR, provided improved diagnostic efficacy and detection sensitivity and also acted as a diffusion barrier for controlled release of cisplatin. The PEG shell provided steric protection and prevented micelles from aggregating. The NR containing polymeric micelles were taken up by Hep G2 cells and localized in the nuclei and cytoplasm. They were non-toxic and had excellent biocompatibility, exhibiting 90% viability at polymer concentrations upto 500 μg/mL. Cisplatin-loaded micelles exhibited a dose-dependent cytotoxic effect on the Hep G2 cells (Bastakoti et al., 2013).

Additional examples of multifunctional drug-loaded micelles are outlined in Table 4.

**Table 4:** **Some examples of multifunctional drug-loaded micelles**.

**Micelle components**	**Drug(s)**	**Targeting ligand**	**Stimulus**	**Imaging moiety**	**References**
P(NIPAAm-*co*-AAm)-b-PCL	DOX	Integrin β4 mAb (recognizes A9 antigen)	Thermo-responsive LCST: 43°C -Magnetic hyperthermia	SPIONs for MRI	Kim et al., 2013
POEGMA-*b*-P(NIPAAm-*co*-NBA-co-Gd)	DOX	–	UV Light	Gd for MRI	Li et al., 2012c
DSPE-PEG, biotin-DSPE-PEG, and lissamine-rhodamine (phospholipid encapsulated SIPP cores) SIPP-PTX micelles	PTX	J591 (against PSMA)	–	Superparamagnetic iron platinum NPs (SIPP) for MRI	Taylor and Sillerud, 2012
PEG-*b*-PCL	DOX	Cetuximab(anti-EGFR mAb)	–	SPIO for MRI	Liao et al., 2011
PEG2000-PE/DC-Chol/DOPE micelles	DOX and DNA	–	–	MnO NPs for MRI	Howell et al., 2013
Folate-poly(ethylene glycol)-*b*-poly[N-(N',N'-diisopropylaminoethyl) glutamine] [folated-PEG-P(GA-DIP)]	DOX	Folate	pH	SPION for MRI	Li et al., 2013c
PEG-*b*-PCL	Auger electron radiotherapy by ^111^In	Trastuzumab-Fab (targets HER2) and 13-mer NLS	–	SPECT/CT imaging by ^111^In label	Hoang et al., 2013
Poly(amidoamine)-poly(L-lactide)-*b*-PEG	DOX	Anti-CD105 mAb (TRC105) and NOTA	pH	^64^Cu for PET imaging	Guo et al., 2013
PEG-*b*-PCL	Sorafenib	Folate		SPIONs for MRI	Zhang et al., 2013

POEGMA-b-P(NIPAAm-co-NBA-co-Gd), Poly(oligo(ethylene glycol) monomethyl ether methacrylate-b-Poly(N-isopropylacrylamide)-co-o-nitrobenzyl acrylate-co-Gd; DOX, Doxorubicin; PTX, Paclitaxel; CREKA, Cys-Arg-Glu-Lys-Ala; NLS, Nuclear localization signal (13 mer peptide CGYGPKKKRKVGG); NOTA, 1,4,7-triazacyclononane-N, N', N-triacetic acid (macrocyclic chelator for ^64^Cu).

## Challenges for the delivery of siRNA and the role of polymeric micelles

Since the discovery of RNAi, there has been an increased interest in developing siRNA based therapies to achieve sequence-specific post-transcriptional silencing of aberrant genes in diseases such as cancer (Fire et al., 1998; Elbashir et al., 2001). RNAi is an endogenous pathway which is utilized by all eukaryotic cells to silence genes post-transcriptionally, and can be triggered by double stranded RNAs (dsRNA) like endogenous microRNA (miRNA), short hairpin RNA (shRNA) and synthetic siRNA (Wang et al., 2010b). The detailed mechanism of the RNAi pathway has been reviewed elsewhere (Hannon, 2002; Rana, 2007; Sashital and Doudna, 2010). Briefly, the dsRNA is processed by the Dicer enzyme into small fragments which are 21–23 nucleotide (nt) base pairs in length (Meister and Tuschl, 2004). The fragments, which possess a sense (passenger) strand and an antisense (guide) strand with respect to the target mRNA, are loaded into the RNA-induced silencing complex (RISC). Here the strands are separated, and the sense strand is cleaved and discarded. The activated RISC-guide strand complex is then directed to the complementary region of the target mRNA to cause it to degrade and prevent its translation (Martinez et al., 2002). Synthetic siRNAs can be introduced into the cell directly and avoid processing by Dicer (Whitehead et al., 2009).

To access and activate the RNAi machinery, the siRNA must be delivered to the cytoplasm of the cell. However, this “delivery of siRNA” poses one of the most formidable challenges to realizing the potential and utility of siRNA therapeutics. Whereas localized targets are accessed directly, the main hurdle is encountered when siRNA is delivered to tissues which are accessed only through systemic administration of agents via the blood (Whitehead et al., 2009). A number of barriers prevent the successful systemic delivery of siRNA. After intravenous administration, naked siRNA exhibits low *in vivo* stability due to quick degradation by nucleases. It has a short half-life due to rapid clearance by the kidneys and uptake by the MPS. The hydrophilic nature and negative charge of siRNAs prevent them from crossing the plasma membrane easily, despite their relatively small size (about 13 kDa) (Liu et al., 2013; Navarro et al., 2013). Other challenges with siRNA include the potential to generate off-target effects due to silencing of genes that have partial homology with the siRNA and engagement of the immune system components to cause immune stimulation (Bumcrot et al., 2006). Moreover, because siRNAs also share the RNAi pathway with endogenous miRNAs, they may compete for the RNAi machinery, saturate it and inhibit normal gene regulation by miRNAs (Kanasty et al., 2012). A number of approaches have been suggested to overcome some of the aforementioned challenges. Chemical modification of the sugars, phosphate linkage or bases of siRNA can increase its stability and also reduce the immune stimulation. Modifications of the 2′ sugar position [2′-fluoro (2′-F), 2′-O-methyl (2′-O-Me) and 2′-O-methoxyethyl (2′-MOE)], locked nucleic acids (LNA) and unlocked nucleic acids (UNA) increase the endonuclease resistance and reduce immunostimulatory activity, whereas introduction of a phosphorothioate (PS) linkage at the 3′ end in the backbone is known to increase stability and resist against exonucleases (Bumcrot et al., 2006). Base modifications are less common than those of sugar or phosphate linkages, but have been employed. The use of 2-thiouracil or pseudouracil can increase the binding specificity and potency and 5-methylation of pyrimidines (using T and 5-Me-C instead of U and C) is also common (Watts et al., 2008). The off-target effects of siRNA and its saturation of the RNAi machinery can be avoided by optimizing the siRNA sequence and structure, and limiting doses of exogenous RNA respectively (Kanasty et al., 2012; Navarro et al., 2013).

The issues of instability in circulation, rapid clearance and short half-life of naked siRNA can be addressed by chemically modifying siRNA or by employing nanocarriers which can protect it from degradation and immune recognition as well as modify its pharmacokinetics favorably *in vivo* (Pecot et al., 2011; Kanasty et al., 2012). The negative charge of siRNA can be masked using cationic carriers which complex the siRNA electrostatically. The positive charge of these carriers also helps in cellular internalization (Musacchio and Torchilin, 2013).

A nanocarrier must possess certain features to be successful as a carrier for systemic siRNA delivery. Ideally, a nanocarrier should: (a) be non-toxic and non-immunogenic, (b) condense siRNA effectively, (c) be stable in the presence of nucleases, (d) protect the siRNA from immune recognition, (e) be large enough to avoid clearance by kidneys, yet small enough to avoid phagocytosis by MPS, (f) avoid non-specific interactions with serum proteins and non-target cells, (g) reach target tissues from the blood and eventually the intracellular compartment, and (h) release the entrapped siRNA efficiently in the cytoplasm to access the siRNA machinery (Daka and Peer, 2012; Kanasty et al., 2013). Polymeric micelles have been used successfully as drug delivery vehicles for the past few decades, which has prompted their use as vehicles for siRNA. Moreover, by engineering micelles with suitable modifications discussed in the previous sections, they may likely meet most criteria for an “ideal” nanocarrier for siRNA.

So far, two main strategies have been used to design polymeric micelles for siRNA delivery. The first involves direct conjugation of hydrophilic (PEG) or hydrophobic (lipid) moieties to siRNA via degradable (e.g., disulfide) or non-degradable linkages, followed by their condensation with polycationic ions to form micellar structures called polyion complex micelles (PICs) or polyelectrolyte complex micelles (PECs). In PIC micelles, the polyion segments are usually made of poly(amino acids) like poly(aspartic acid) or poly(L-lysine) or PEI (Oishi et al., 2005; Kim et al., 2008; Suma et al., 2012). In the second strategy, siRNA is complexed with an amphiphilic block copolymer containing polycation (or lipid) segment followed by micellization of the block copolymer-siRNA complex (Falamarzian et al., 2012; Navarro et al., 2013). Nanoparticles including polymeric micelles enter cells by endocytosis (Decuzzi and Ferrari, 2008). One of the major intracellular barriers for siRNA delivery is that of endosomal escape following its delivery by various carriers. Following endocytosis, the siRNA-loaded carriers in membrane-bound endocytic vesicles fuse with early endosomes to become increasingly acidic as they mature into late endosomes (pH 5–6). Finally the endosomal contents are delivered to the lysosome, where the pH drops further (pH ~4.5), and where hydrolysis of proteins and nucleic acids take place (Dominska and Dykxhoorn, 2010; Singh et al., 2011). To avoid lysosomal degradation, it is essential for the siRNA to escape the endosome, be released into the cytosol and interact with the RNAi machinery (Dominska and Dykxhoorn, 2010). To overcome this “endosomal escape barrier,” polymeric micelles can be designed to incorporate cationic polymers such as PEI which act as “proton sponges” to disrupt the endosomes and release siRNA in the cytosol. Alternatively, pH-responsive polymers can be used to construct polymer micelles, so that they disrupt and release the siRNA at the endosomal pH. Finally fusogenic lipids, cell penetrating peptides, other polymers with high buffering capacity and photosensitizers (upon light activation they induce endosomal disruption via singlet oxygen production) can be used to engineer polymeric micelles to overcome the issue of endosomal escape (Dominska and Dykxhoorn, 2010). In the sections that follow, we discuss some examples of multifunctional micelles from the recent literature which have been used to deliver either siRNA alone or siRNA in combination with drugs.

### Multifunctional micelles for delivery of siRNA

Many interesting micelle-forming amphiphilic block copolymers have been developed for siRNA delivery over the past few years. These basic platforms are being modified continuously to achieve maximum benefit from them, by introducing targeting ligands or incorporating environmentally-sensitive blocks or links within them. For all siRNA delivery platforms including micelles, a major focus is on preventing siRNA degradation from the time it is introduced in the systemic circulation, until it reaches the RNAi machinery in the cytoplasm, after navigating the endocytic pathway for intracellular trafficking and its subsequent endosomal escape.

Christie et al. reported the development of multifunctional micelles for siRNA delivery formed from the stable assembly of siRNA with block copolymers possessing three main features: a siRNA binding segment containing thiols, a hydrophilic non-binding segment and a cell-surface binding peptide (Christie et al., 2012). The block copolymer used was PEG-b-poly(_L_-lysine) (PEG-b-PLL) containing lysine amines modified with 2-iminothiolane (2IT). Building on their previous work where the RNAi activity of the micelles formed using this copolymer was found to be low (Matsumoto et al., 2008), the authors further modified the block copolymer with cyclo-Arg-Gly-Asp (cRGD) peptide at the PEG terminus to enhance tumor accumulation, cell uptake and sub-cellular distribution. The 2IT modification of the lysine amines introduced amidines and free thiols into the lysine segment of the block copolymer, which increased the stability of micelles through disulfide cross-linking in the core. It also imparted micelles with a site-specific siRNA release function in response to the highly reducing environment within cells. The 2IT modification also had a micelle-stabilizing effect due to the formation of cyclic-N-substituted 2IT ring structures in the lysine side chains. Electrostatic interactions between oppositely charged macromolecules resulted in charge neutralization and self-assembly to form micelles, with siRNA incorporated into the micelle core and PEG forming the shell. The cRGD-targeted micelles improved siRNA delivery both in *vitro* and *in vivo*. Micelles incorporating siRNAs against VEGF (to target tumor mass) and those incorporating siRNA against vascular endothelial growth factor receptor 2 (VEGFR2) (to target blood vessel endothelial cells) were prepared. The siRNA-loaded, cRGD-modified micelles enhanced the gene silencing ability, improved cell uptake, and had better sub-cellular distribution *in vitro.* They also improved accumulation in the tumor mass and tumor-associated blood vessels following i.v. injection in mice. They also effectively inhibited growth of subcutaneous HeLa-Luc tumors and silenced genes in the tumor mass following treatment with antiangiogenic siRNAs. No tumor growth reduction was observed with naked siRNA, micelles lacking the cRGD peptide, or those without 2IT-modified lysines, which was consistent with *in vitro* results (Christie et al., 2012). TAT, another cell-penetrating peptide was conjugated via a disulfide bond to an amphiphilic block copolymer mPEG-PCL and evaluated for siRNA delivery both *in vitro* and *in vivo* (Kanazawa et al., 2012). The MPEG-PCL-SS-TAT/siRNA micelles showed a significantly higher intracellular uptake of 6-carboxyfluorscein-aminohexyl (FAM)-siRNA than naked FAM-siRNA and an uptake equivalent to the positive control LipoTrust at a nitrogen to phosphate (N/P) ratio of 30. The micelles without siRNA (MPEG-PCL-SS-TAT) did not induce substantial cytotoxicity in S-180 sarcoma cells at any of the reported N/P ratios. The MPEG-PCL-SS-TAT/anti-VEGF siRNA micelle complexes were evaluated *in vivo* in S-180 sarcoma tumor-bearing mice. Relative to control mice and to groups injected with naked VEGF siRNA or control siRNA bearing micelles, the tumor volumes were significantly suppressed for the MPEG-PCL-SS-TAT/siVEGF group, which correlated with the reduction in VEGF secretion from these tumors. The suppressed VEGF secretion was attributed to improved siRNA release in the cytosol after cleavage of the S-S bond by intracellular GSH, which enables release of TAT and the dissociation of siRNA from micelles (Kanazawa et al., 2012).

**Figure 7:**
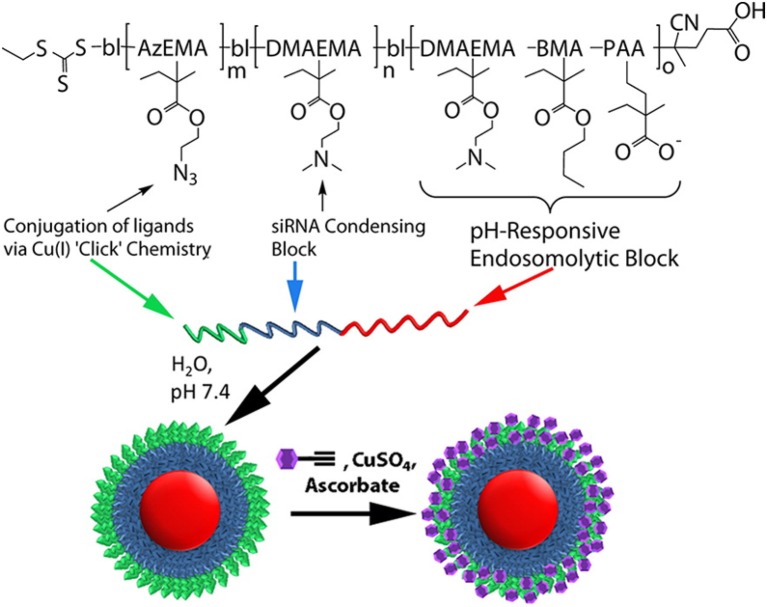
**Smart polymeric nanoparticles for mannose receptor-targed cytosolic delivery of siRNA**. Schematic representation of the triblock copolymers and formulation into multifunctional nanoscale siRNA delivery vehicles. The blocks include a pH-responsive block that is capable of disrupting endosomes at low pH (red), a cationic block for condensation of nucleic acids (blue), and an azide-displaying block (green) for conjugation of targeting motifs (purple) via click chemistry. Reprinted with permission from Yu et al. (2013b). Copyright © 2013 American Chemical Society.

Significant levels of resident macrophages have been observed in many cancers, which have been correlated with poor prognoses. Tumor associated macrophages thus represent an interesting target for cancer therapy (Lewis and Pollard, 2006). However, the delivery and cytoplasmic release of siRNA in macrophages is a challenging task, due to their high degradative potential. Yu et al. reported pH-responsive polymeric micelles which were mannosylated using “click” chemistry to allow CD206 (mannose receptor)-targeted siRNA delivery to tumor associated macrophages which show an up-regulation of these receptors (Yu et al., 2013b). The mannosylated micelles had been incorporated with various functions in their polymer blocks and were synthesized in three stages: (I) Sequential reverse addition-fragmentation chain transfer (RAFT) polymerization and purification was first used to synthesize the polymeric components, (II) Alkyne functionalized mannose was synthesized separately, and (III) The polymers from stage (I) were formed into micelles and reacted with alkyne functionalized mannose from stage (II) using alkyne-azide click chemistry which immobilizes mannose on the micelle corona. The micelle blocks consisted of a pH-responsive, core-forming terpolymer capable of disrupting endosomes at low pH (butyl methacrylate-co-2-propyl acrylic acid-co-2-dimethylaminoethyl methacrylate) (BMA-co-PAA-co-DMAEMA), a cationic block for condensing siRNA (DMAEMA) and an azide-presenting corona-forming block for the attachment of alkyne-functionalized mannose (2-azidoethyl methacrylate (AzEMA) (Figure 7).

The resulting triblock copolymers poly(BMA-co-PAA-co-DMAEMA)-*b*-poly(DMAEMA)-*b*-poly(AzEMA) self-assembled into micelles in aqueous media at pH 7.4. The mannosylated micelle nanoparticles (ManNPs) had a four-fold improved siRNA delivery into macrophages compared with non-targeted carriers and achieved 87 ± 10% knockdown of a model gene in primary macrophages following a 24 h treatment. They preferentially delivered siRNA into immortalized human macrophages (13-fold higher) relative to model breast cancer cell lines. The ManNPs as well as diblock copolymers without the targeting agent were both capable of escaping the endosomal compartment in a pH-dependent manner as confirmed by the red blood cell (RBC) hemolysis assay, a function conferred by the core-forming, pH-responsive, endosomolytic terpolymer block (Yu et al., 2013b). The same pH-responsive terpolymer block was previously reported by Palanca-Wessels et al. in another multifunctional micellar system for CD22 receptor-targeted delivery of siRNA to lymphoma cells. The CD22-targeted polymeric micelles were effective at a low dose of 15 nmol/l siRNA and produced a 70% reduction in glyceraldehyde-3-dehydrogenase (GAPD) gene expression in DoHH2 lymphoma cells (Palanca-Wessels et al., 2011). Leroux and co-workers also reported pH-responsive, core-shell type PIC micelles (PICMs) decorated with an antibody fragment directed against the transferrin receptor (anti-CD71) for delivery of siRNA (Felber et al., 2011). The micelles were prepared by complexing poly(ethylene glycol)-*b*-poly(propyl methacrylate-co-methacrylic acid) (PEG-*b*-P(PrMA-co-MAA) with different polyamidoamine (PAMAM) dendrimers and nucleic acids to form the PICMs. Under mildly acidic conditions found within the endosomal compartment, the PICMs lose their shell to release the PAMAM/nucleic acid core due to protonation of MAA units. The micelles were stable in serum and protected siRNA from degradation. Cell uptake studies with PC-3 (prostate cancer) cells using flow cytometry revealed a significantly higher uptake for the anti-CD71 Fab'-PICMs when compared to native PICMs and non-specific antibody-bearing micelles. The targeted, siRNA-loaded PICMs down-regulated the expression of the anti-apoptotic oncoprotein Bcl-2 *in vitro*, when using either the unmodified or 2′-modified (2′F-RNA and 2′F-ANA) sequences. The chemically modified siRNA however required a five-fold lower concentration (10 vs. 50 nM to achieve the same silencing as the unmodified siRNA (Felber et al., 2011).

Additional examples for multifunctional siRNA-loaded micelles are given in Table 5.

**Table 5:** **Examples of multifunctional siRNA-loaded micelles**.

**Micelle forming components**	**siRNA**	**Targeting ligand**	**Stimulus**	**References**
PEO-*b*-PCL	MDR1 siRNA	RGD4C (targets α_v_β_3_)/TAT (CPP)	–	Xiong et al., 2010
PEG-*b*-poly(_*L*_-lysine)-*g*-(ss-lPEI)	XIAP (anti-apoptotic) siRNA	Herceptin (targets Her2/neu)	Redox (disulfide bonds)	Li et al., 2014
PEG-*b*-P(PrMA-co-MAA) shell and PAMAM core PIC micelles	Bcl-2 siRNA	Anti-CD71 Fab'(targets transferrin receptors	pH	Elsabahy et al., 2009
6 arm PEG-Hph1/cl KALA PECs	GFP or VEGF siRNA	Hph1 (CPP)	Redox	Choi et al., 2010
PDMAEMA-*b*-PDPAEMA (Amphotericin B loaded in PDPAEMA core)	GL3 luciferase siRNA	–	Dual pH (amphotericin B caused endosomal escape via membrane destabilization)	Yu et al., 2011
mPEG-*b*-PCL and PCL-*b*-PPEEA mixed micelles	Apolipoprotein B siRNA (hepatocyte specific)	N-galactosamine (targets ASGPr)	–	Wang et al., 2013

lPEI, Low mol weight polyethyleneimine; PEG-b-P(PrMA-co-MAA), poly(ethylene glycol)-block-poly(propyl methacrylate-co-methacrylic acid); PAMAM, polyamidoamine; cl KALA peptide, cross-linked CWEAKLAKALAKALAKHLAKALAKALKAC; Hph1, cell penetrating peptide (YARVRRRGPRR); PDMAEMA-b-PDPAEMA, poly(2-(dimethylamino)ethyl methacrylate)-block-poly(2-(diisopropylamino)ethyl methacrylate); ASGPr, asialoglycoprotein receptor; PPEEA, poly(2-aminoethyl ethylene phosphate.

### Multifunctional micelles for the combined delivery of drugs and siRNA

siRNA therapy using polymeric micelles has shown considerable promise and is being investigated widely. However, tumors are highly prone to genetic mutations, which may hinder the effectiveness of siRNA as a single agent in the treatment of malignancies (Liu et al., 2013). Moreover, conventional anti-cancer agents also suffer from limitations like off-target effects and multi-drug resistance (MDR), both of which hamper cancer therapy significantly. Efforts to develop molecules which inhibit the function of the drug transporter proteins like P-glycoprotein (Pgp) (encoded by the MDR1 gene) to sensitize tumor cells to anti-cancer agents have met with limited clinical success so far, due to the non-specific nature of these inhibitors (Shukla et al., 2008). In such cases, employing RNAi to down-regulate the expression of MDR genes to specifically inhibit Pgp expression rather than merely its function, followed by conventional chemotherapy could reap greater benefits for cancer therapy (Wu et al., 2003). A number of studies have reported that pre-treatment of cancer cells with siRNAs followed by conventional anticancer drugs can sensitize the cells significantly toward the drug and make therapy more effective (Spankuch et al., 2007; Macdiarmid et al., 2009; Zhang et al., 2011a; Salzano et al., 2014). However, to gain the maximum effect from both siRNA and drug *in vivo*, they must be delivered simultaneously to the same tumor cell following systemic administration and, ideally, distribute within cells at an optimized ratio for maximal cooperation (Sun et al., 2011b). In this section, we discuss some examples of multifunctional polymeric micelles which incorporate siRNA as well as drug within the same nanocarrier.

**Figure 8:**
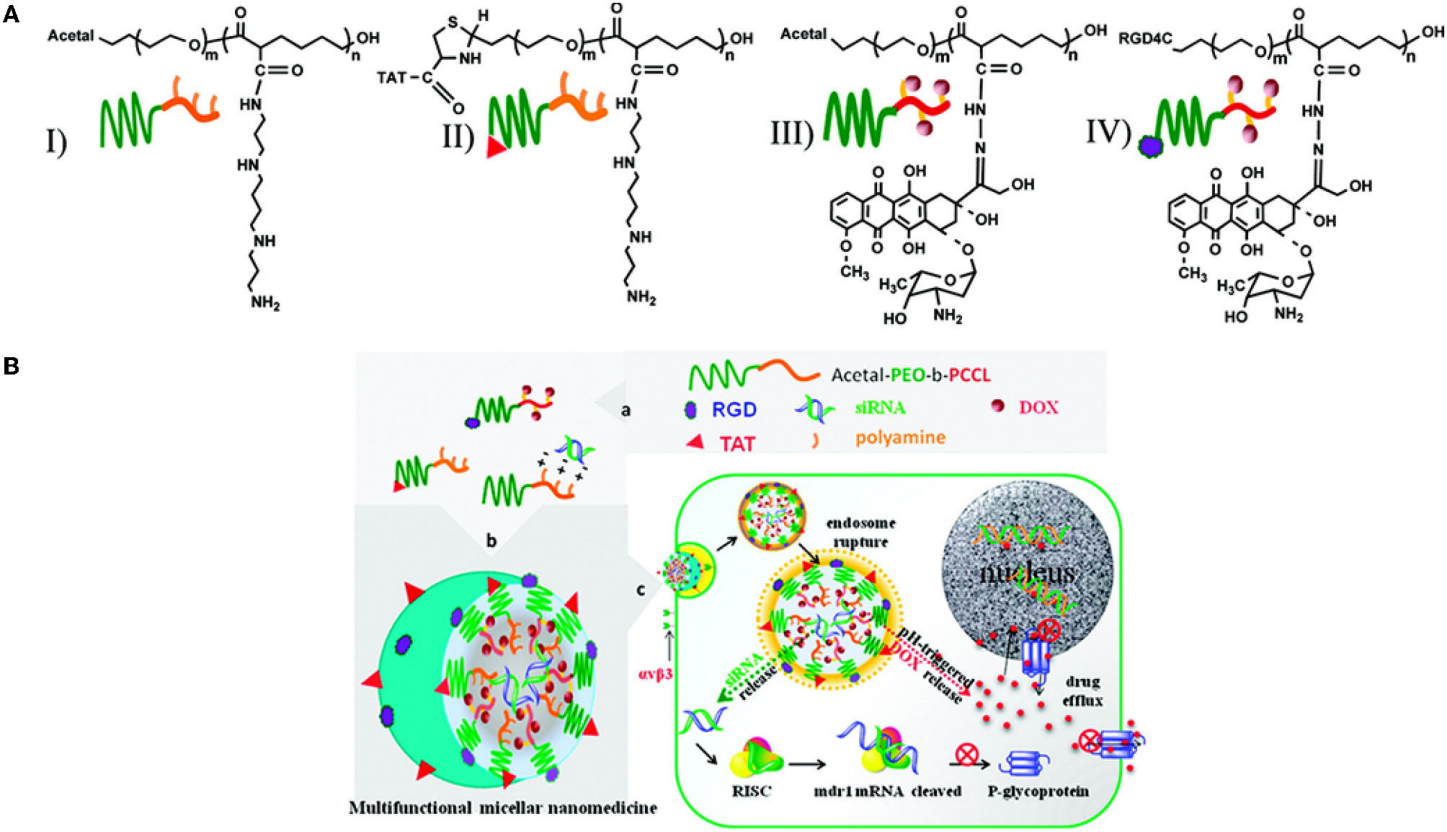
**(A)** Schematic illustration of acetal- and TAT-PEO-*b*-P(CL-g-SP) (I and II) and acetal- and RGD4C-PEO-*b*-P(CL-Hyd-DOX) (III and IV). **(B)** Rational design of a multifunctional micellar nanomedicine for targeted co-delivery of siRNA and DOX to overcome multidrug resistance. **(a)** Chemical structure of functionalized copolymers with ligands at the end of the PEO block and conjugated moieties on the PCL block. **(b)** Assembly of multifunctional micelles with DOX and siRNA in the micellar core and RGD and/or TAT on the micellar shell. **(c)** Schematic diagram showing the proposed model for the intracellular processing of targeted micelles in MDR cancer cells after receptor-mediated endocytosis, leading to cytoplasmic siRNA delivery followed by P-gp down-regulation on the cellular and nuclear membrane and endosomal DOX release, followed by DOX nuclear accumulation. Reprinted with permission from Xiong and Lavasanifar (2011). Copyright © 2011 American Chemical Society.

Multifunctional micelles composed of PEO-b-PCL block copolymers were reported with functional modifications on both the blocks (Xiong and Lavasanifar, 2011). These micelles could co-deliver siRNA and DOX, enable passive and active targeting, provide for cell membrane translocation and provide a pH-triggered drug release in the endosomes. The PCL core of the micelles incorporated short polyamines (spermine (SP)) to complex MDR1 siRNA, conjugated DOX chemically via a pH-sensitive hydrazone linkage, and they could also conjugate fluorescent imaging probes to track micelles *in vitro and in vivo*. To the virus-like shell of these micelles, two ligands were attached: an active targeting ligand, RGD4C specific for integrin (αvβ 3) receptors and a cell penetrating TAT-peptide to facilitate the intracellular uptake (Figure 8).

The final micelle formulation was obtained by mixing plain or peptide-modified PEO-b-P(CL-g-SP)/siRNA and PEO-*b*-P(CLHyd-DOX) block copolymers. The micelles were taken up by the cells through receptor-mediated endocytosis, and released siRNA in the cytoplasm due to endosomal rupture facilitated by spermine and TAT peptide. The traceable micelles were prepared by conjugating a near-infrared fluorophore (Cy5.5) (NIRF) to the spermine side chain or by using fluorescently labeled (Dy677) siRNA which allowed tracking of both the carrier as well as incorporated siRNA *in vivo*. The RGD/TAT-micelles containing MDR1-siRNA demonstrated significant cellular uptake, improved penetration and enhanced the cytotoxicity of DOX in DOX-resistant cells. The cytotoxicity was a result of down-regulation of P-gp expression on cell and nuclear membranes caused by cytoplasmic delivery of siRNA and DOX (Xiong and Lavasanifar, 2011).

**Figure 9:**
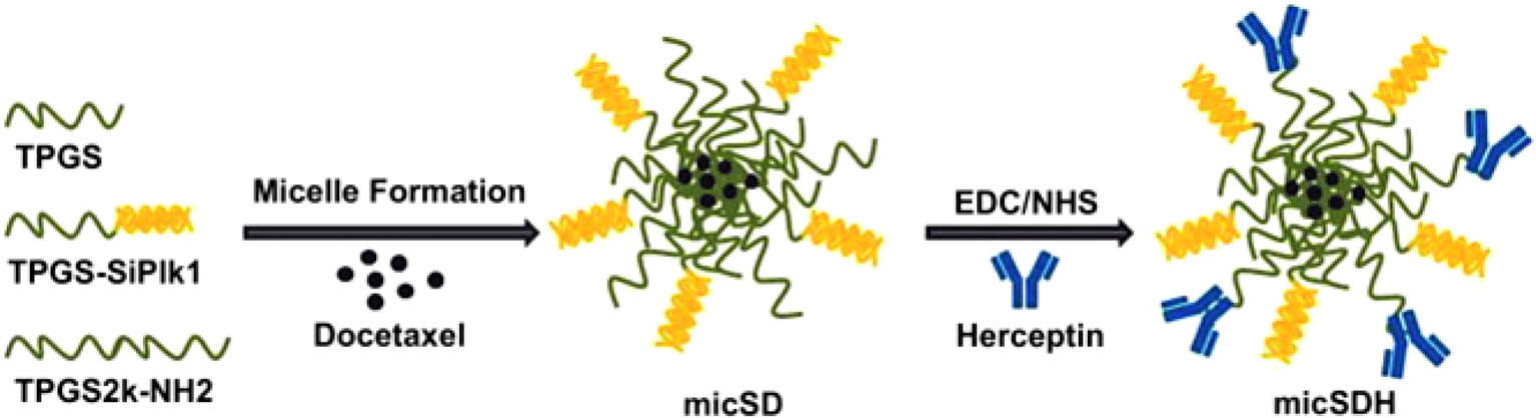
**Schematic illustration of formulation of the docetaxel loaded TPGS–siPlk1/TPGS micelles (micSD) and the herceptin-conjugated docetaxel loaded TPGS–siPlk1/TPGS micelles (micSDH)**. Reprinted from Zhao et al. (2013).

Zhao et al. reported on multifunctional micelles capable of co-delivering docetaxel as well as siRNA against polo-like kinase 1 (Plk1), which is over-expressed in a number of tumors and plays a crucial role in cell mitosis (Zhao et al., 2013). The authors conjugated siPlk1 to D-α-tocopheryl polyethylene glycol succinate (vitamin E TPGS or TPGS), a water soluble vitamin E derivative, using a disulfide bond to form TPGS-siPlk1. The disulfide bond was susceptible to high intracellular GSH concentrations, which caused the release of siPlk1 and also accelerated drug release due to reduced stability of micelles following cleavage of the disulfide bond. To develop Herceptin-conjugated micelles, a mixture of TPGS-siPlk1conjugate and TPGS or amine terminated TPGS (TPGS-NH_2_) was used at a designated ratio for co-delivery of siRNA and docetaxel (Figure 9).

The micelles were evaluated *in vitro* in cells expressing low (NIH3T3), moderate (MCF-7) or high levels of HER2 (SK-BR-3). To functionalize micelles with Herceptin, TPGS-NH_2_ was used instead of TPGS followed by Herceptin conjugation via EDC-NHS chemistry. The siPlk-1 and Herceptin modified micelles successfully internalized into the cytoplasm of SK-BR-3 cells. Moreover, the Herceptin modification enhanced the therapeutic efficacy of micelles due to its inherent toxicity to cancer cells as well as its ability to undergo receptor mediated endocytosis and assist the nanocarrier's entry into the cytoplasm. The Herceptin modified micelles showed significantly higher cellular uptake and low IC50 values in SK-BR-3 cells compared to micelles not modified with Herceptin (Zhao et al., 2013).

Another study also reported the delivery of siRNA against Plk-1 in combination with DOX to sensitize ovarian cancer cells (NCI/ADR-RES) to DOX (Benoit et al., 2010). The cationic micelles were formed from diblock copolymers of dimethylaminoethyl methacrylate (pDMAEMA) and butyl methacrylate (BMA). The butyl core was responsible for micelle formation while the siRNA condensation was facilitated by the positively charged p(DMAEMA). A pH-responsive endosomolytic copolymer of poly(styrene-*alt*-maleic anhydride) (pSMA) was complexed to the positively charged siRNA/micelle to form a ternary complex by electrostatic interaction. DOX was loaded in the micelle cores to demonstrate simultaneous dual delivery with siRNA from a single carrier. However, for this particular study, the authors found that with dual delivery the effects on caspase activation and cell toxicity were weaker than those obtained with singly loaded siRNA ternary complexes due to a limitation in DOX loading concentration (0.2 μg/ml) (Benoit et al., 2010).

**Figure 10:**
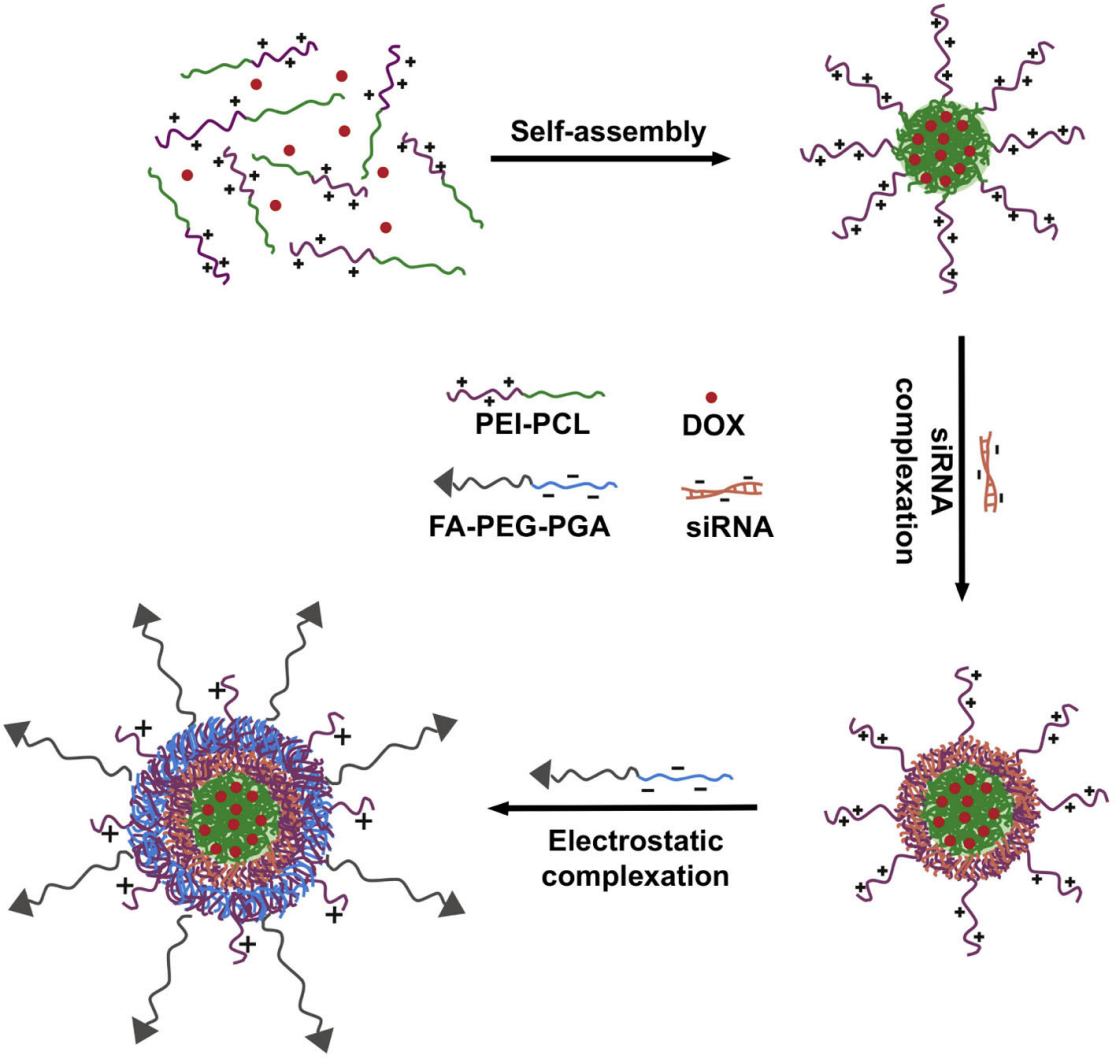
**Formation of hierarchical nano-assemblies for combinatorial delivery of siRNA and anticancer drugs**. Reprinted from Cao et al. (2011).

Cao et al. synthesized diblock copolymers of linear PEI and PCL (PEI-PCL) which self-assembled into cationic biodegradable polymeric micelles (Cao et al., 2011). Furthermore, the micelles were able to load and co-deliver anti-apoptotic Bcl-2 siRNA and DOX and utilized folic acid as a targeting agent for human hepatic cancer cells Bel-7402 (Figure 10). To incorporate folic acid, it was first conjugated to a polyion, poly(ethylene glycol)-block-poly(glutamic acid) (FA-PEG-PGA) and then coated electrostatically onto the surface of cationic PEI-PCL micelles preloaded with siRNA and DOX. This multifunctional hierarchial nano-assembly was capable of simultaneous delivery of drug and siRNA in a targeted manner to yield a synergistic effect of RNAi and chemotherapy on cancer targets.

The approach for incorporation of folate was relatively simple, avoided the toxicity associated with cationic carriers and did not affect siRNA complexation. At certain ratios of PEI-PCL nitrogen-to-siRNA phosphate (N/P) and FA-PEG-PGA carboxyl-to-PEI-PCL amine (C/N), the micelles demonstrated high transfection efficiency as well as controlled release of DOX. The folate-targeted delivery of anti-apoptotic Bcl-2 siRNA resulted in significant gene suppression at both the mRNA and protein expression levels compared to the non-targeted micelles. The suppression of DOX-inducible up-regulation of the anti-apoptotic Bcl-2 gene led to enhanced cell apoptosis in Bel-7402 cells and potentiated the effect of DOX in inducing cell death through a synergistic effect of siRNA and DOX (Cao et al., 2011). Following up on these promising *in vitro* results, the authors extended their research to determine if this multifunctional nanoplatform would show a synergistic effect *in vivo*, and to elucidate the molecular mechanism of the synergistic effect (Cheng et al., 2012). A rat model with an *in situ* C6 glioma implant was used for *in vivo* studies. In the *in vitro* studies the folate-targeted multifunctional micelles induced significant cell apoptosis in C6 cells even at a low dose of DOX (0.5 μg/mL) compared to free DOX, which caused apoptosis only at high doses (15 μg/mL). Molecular investigations showed that the targeted nanocarriers effectively suppressed the anti-apoptotic response induced by DOX, and sensitized C6 cells to DOX treatment both *in vitro* and *in vivo*. In the animal studies, the folate-targeted co-delivery of Bcl-2 siRNA and DOX caused a significant down-regulation of the Bcl-2 gene and also up-regulated the pro-apoptotic Bax gene, which increased the activated caspase-3 levels significantly, resulting in cell apoptosis in the tumor tissues. The targeted co-delivery strategy led to a synergistic effect *in vivo* causing effective tumor growth inhibition as well as prolonged survival time over treatment with micelles with single agents or non-targeted micelles (Cheng et al., 2012). Zou et al. from the same group reported a triblock copolymer PEG-PEI-PCL instead of PEI-PCL mentioned above and conjugated it to FA. This copolymer self-assembled to form cationic micelles which could then complex Bcl-2 siRNA. These micelles simultaneously delivered siRNA and DOX with successful results *in vitro* in SKOV-3 ovarian cancer cells. This ternary copolymer complex was reported to have better stability than that formed using the hierarchial multilayer assembly where the PEG coating was achieved by electrostatic interaction rather than covalent linkage (Zou et al., 2012).

In a recent study, Zhu et al. reported the development of MMP-2 sensitive multifunctional polymeric micelles for the co-delivery of siRNA (anti-survivin or anti-GFP) and PTX (Zhu et al., 2014). They developed a simple MMP-2-sensitive self-assembling copolymer, PEG-pp-PEI-PE using a synthetic octapeptide (GPLGIAGQ) which was also utilized in their previous investigations with both, liposomes and micelles for MMP-2-sensitive tumor targeting (Zhu et al., 2012, 2013). The micelles exhibited efficient down-regulation of the reporter gene (GFP) in GFP expressing cells (copGFP A549) and survivin in PTX-resistant non-small cell lung cancer cells (A549 T24). The PEG-pp-PEI-PE/PTX micelles significantly increased the cytotoxicity of PTX in both PTX-sensitive (A549) and resistant (A549 T24) cells relative to the free drug or non-sensitive micelles. The simultaneous delivery of anti-survivin siRNA and PTX resulted in a synergistic effect, significantly reducing the IC_50_ of PTX to 15 nM (from 96 nM for free PTX). *In vivo*, although the co-delivery efficacy for siRNA and PTX was not as pronounced as that under *in vitro* conditions, the MMP-2 sensitive micelles showed a 2.4-fold higher internalization of siRNA and PTX compared to the non-sensitive micelles due to the de-shielding of PEG and exposure of PEI (Zhu et al., 2014).

## Conclusions and future directions

The last several years have seen rapid advances in the use of polymeric micelles for delivery of a variety of cargoes from conventional anti-cancer drugs to biological macromolecules such as DNA, siRNA, antibodies and oligonucleotides. Chemical modifications in the structure of micelle-forming block copolymers have enabled the development of sophisticated micelles which combine multiple modalities within a single carrier. There is a clear shift from developing just single-therapeutic agent loaded micelles to micelles which combine more than one type of therapeutic payload and which can also be modified for active targeting, delivery of imaging agents and response to special cues provided either by the tumor microenvironment or externally, to spatially and temporally control the release of entrapped cargoes.

This review has discussed a number of examples which cover a wide range of polymeric micelle modifications—from polymeric micelles which are modified for passive targeting and rely on the EPR effect, to progressively more complex systems which incorporate targeting ligands, respond to various stimuli and finally to systems which simultaneously and seamlessly incorporate multiple modifications as well as combinations of drugs and biologics like siRNA to give rise to multifunctional polymeric micelles. We may certainly look to the future of polymeric micelles with a lot of optimism, given their inherent advantages and ease of introducing structural modifications. In part, this is supported by the wide variety of amphiphilic copolymers available for manipulation, better control of micelle characteristics and clinical success with passively targeted polymeric micelles for anti-cancer drugs. However, as far as siRNA delivery is concerned, much work remains before polymeric micelle therapeutics can be successfully translated into clinical usage. This stems from the inherent difficulty in delivering siRNA and a host of biological barriers encountered en route to its ultimate destination, the RNAi machinery in the cytoplasm. There exist certain established criteria for the successful development of polymeric micelles for siRNA delivery based on the previous as well as ongoing investigations. These include controlling the micelle size to be large enough to preclude renal filtration but small enough to avoid phagocytosis, chemical modifications of siRNA to improve stability against nuclease degradation and avoid immunostimulation, PEGylation to prevent rapid elimination, non-specific interactions and to evade immune surveillance, use of polycations in micelle blocks to effectively condense siRNA and improve transfection, incorporation of endosomolytic agents to assist the endosomal escape of siRNA, and finally the use of targeting ligands to improve uptake by specific cells (Kanasty et al., 2013). However, to arrive at an efficacious micellar formulation for siRNA, it is critical to optimize each of these parameters so that enhancing one of them does not adversely affect the other. For example, polycations enhance the condensation of siRNA in the micelles and improve its transfection efficacy, but may negatively affect the overall safety of the micelles *in vitro and in vivo*, which necessitates the use of low molecular weight polycations that are relatively safer to use. To date, many oncogenic targets including those involved in apoptosis, drug resistance, proliferation and angiogenesis have been investigated for siRNA-mediated therapy of cancer. However, the safe and efficient delivery of siRNA into target cells still presents a formidable challenge (Liu et al., 2013). We must address key challenges in siRNA delivery to optimize micelle formulations to enable their translation into clinically acceptable therapeutics. There is a need to develop polymers which allow efficient siRNA loading and protection within the formulation without the accompanying adverse effects. Efforts need to be focused on development of more stable micelle formulations with siRNA that allow long term storage if they are to eventually reach the clinics. *In vivo* safety issues like immune stimulation and off-target effects of both siRNA and micelle forming materials need to be given serious consideration as well. The PK/PD parameters and biodistribution of siRNA after systemic administration must be studied systematically to arrive at optimal siRNA dosing regimens. Finally, studies must also be undertaken to investigate optimized ratios of drug and siRNA when loaded simultaneously in the same carrier to ensure their synergistic therapeutic effect (Liu et al., 2013).

Multifunctional micelles have gained immense popularity because of their versatility in simultaneously incorporating a variety of different payloads (therapeutic and imaging) and their ability to withstand multiple modifications (active and passive targeting, response to stimuli, imaging) to enable cancer cell specific targeting and therapy. In the light of what has been discussed, we can consider these “smart” polymeric micelles as our best current option for delivery of nucleic acid therapeutics, in particular, siRNA, albeit not without certain limitations. It is crucial to ensure that the functionalities incorporated within a polymeric micelle function seamlessly in perfect coordination with each other. With many different functions and modifications, the architecture of the micelles becomes more complex which can lead to difficulties in their reproducible synthesis and scale-up for manufacture. Impediments to clinical translation may result from the challenge in developing a robust manufacturing process, its cost effectiveness, and finally the regulatory requirements that have to be met when introducing a complex nanocarrier in the clinic. Incorporating siRNA along with drugs and various ligands within a multifunctional micelle may prove to be technically challenging on a large scale.

In spite of the evident hurdles, there are a number of elegantly designed multifunctional micellar formulations under active investigation, and as new technologies develop, there will be more data on such multifunctional platforms for further research. It is a well-known fact that the translational potential of a system increases if it has a simplified design and ease of development. To that end, polymeric micelles, like their nanocarrier counterparts, liposomes; are already well ahead of other nanocarriers in terms of proven clinical success. The challenge then remains to harness the success with passively targeted micelles to design elegant multifunctional polymeric micelles capable of delivering multiple therapeutic molecules simultaneously.

### Conflict of interest statement

The authors declare that the research was conducted in the absence of any commercial or financial relationships that could be construed as a potential conflict of interest.
